# GD3 synthase drives resistance to p53-induced apoptosis in breast cancer by modulating mitochondrial function

**DOI:** 10.1038/s41388-025-03432-x

**Published:** 2025-05-17

**Authors:** Vivek Anand, Fouad El-Dana, Natalia Baran, Jenny Borgman, Zheng Yin, Hong Zhao, Stephen T. Wong, Michael Andreeff, V. Lokesh Battula

**Affiliations:** 1 Section of Molecular Hematology and Therapy, Department of Leukemia, The University of Texas MD Anderson Cancer Center, Houston, TX USA; 2 Department of Hematology and Central Hematological Laboratory, Bern University Hospital, University of Bern, Bern, Switzerland; 3 Department of Systems Medicine and Bioengineering, Houston Methodist Neal Cancer Center, Weill Cornell Medicine, Houston, TX USA; 4 Department of Breast Medical Oncology, The University of Texas MD Anderson Cancer Center, Houston, TX USA; 5 Department of Internal Medicine, Massey Comprehensive Cancer Center, Virginia Commonwealth University, Richmond, VA USA

**Keywords:** Breast cancer, Mechanisms of disease

## Abstract

*TP53* mutations are common in breast cancer (BC) and are associated with poor prognosis. GD3 synthase (GD3S/*ST8SIA1*), a gene associated with breast cancer stem cells, is upregulated in tumors with p53 mutations. However, the functional relationship between GD3S and p53 is unknown. Here, we show that GD3S levels are highest in breast tumors with specific p53 mutations. Functional studies revealed that wild-type (WT) p53 inhibits GD3S expression, whereas mutation in p53 enhances GD3S expression by upregulating GD3S promoter activity. Moreover, we found that GD3S inhibits wild-type p53–induced apoptosis in BC cells, while BC cells harboring gain-of-function p53 mutations are dependent on GD3S for their growth. Mechanistic insights indicate that GD3S strengthens mitochondrial function by regulating their oxygen consumption rate and membrane polarity. Our findings demonstrate that specific GOF p53 mutations rely on GD3S to exert their tumor-promoting effects and that GD3S is a novel anti-apoptotic factor in BC cells.

**Figure:**
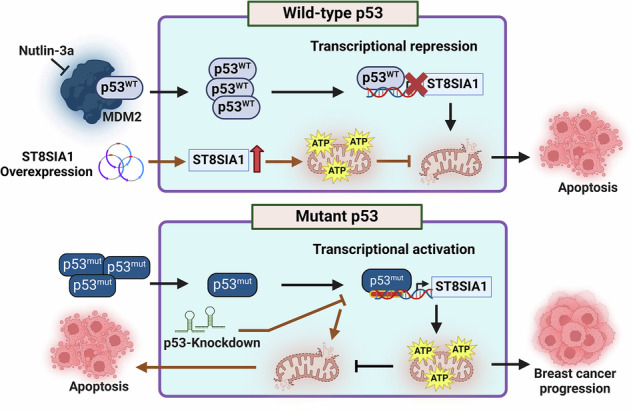
Stabilizing WT p53 and reducing mutant p53 levels downregulates GD3S expression, thereby augmenting apoptosis. GD3S overexpression counteracts the cell death triggered by WT p53 stabilization in BC cells, as well as that triggered by p53 knockdown in cells with specific GOF p53 mutations, which suggests that GD3S helps confer apoptosis resistance.

Stabilizing WT p53 and reducing mutant p53 levels downregulates GD3S expression, thereby augmenting apoptosis. GD3S overexpression counteracts the cell death triggered by WT p53 stabilization in BC cells, as well as that triggered by p53 knockdown in cells with specific GOF p53 mutations, which suggests that GD3S helps confer apoptosis resistance.

## Introduction

The tumor suppressor protein p53 is essential for maintaining genomic stability and regulating cellular responses to stress. Mutations in p53 are common in breast cancer and especially prevalent in triple-negative BC (TNBC); up to 80% of TNBCs have p53 mutations that are associated with poor prognosis [1]. Recent efforts aimed at therapeutically targeting mutant p53 in human malignancies, including BC, have yielded only limited success in early-phase clinical trials. This is attributed to a range of factors encompassing the diverse cellular functions of p53, its structural complexity, and the heterogeneous array of mutations in the p53 gene [2, 3]. Developing effective p53-targeting therapies for BC requires an improved understanding of the molecular mechanisms underlying the association between p53 mutations and the genes responsible for BC progression.

We previously reported that ganglioside GD2 expression identifies BC stem cells and is associated with increased tumorigenesis [4–8]. Interestingly, GD3 synthase, a key regulatory enzyme that catalyzes the rate-limiting step of GD2 biosynthesis, is significantly upregulated in BC patients with p53 mutations [7]. Knockdown of this enzyme prevents tumor initiation in mouse models [7]. Like p53 expression, GD3S expression is regulated by environmental stress; we have shown that metabolic stress induces the cancer stem cell phenotype in BC cells via upregulation of GD3S [5]. However, the link between p53 and GD3S and their combined role in tumorigenesis has not been elucidated.

Various forms of p53 mutations have been observed to influence the regulation of cancer stem cell functions [9–11]. These mutations have been broadly categorized as loss-of-function (LOF) or gain-of-function (GOF) mutations [12]. While LOF mutations disarm p53’s tumor-suppressing functions like DNA repair, cell-cycle arrest, and apoptosis, GOF mutations endow p53 with oncogenic properties [9, 12]. Often associated with GOF phenotypes are hotspot p53 mutations, most frequently missense mutations predominantly impacting the DNA-binding domain of p53 and promoting tumor progression [9–15]. The frequent co-occurrence of GD3S in GD2^+^ tumors with different p53 mutations, including hotspot mutations, as well as GD3S’s relationship with wild-type p53, suggests collaborative and complementary functions of GD3S and p53 in driving tumor progression, but these remain poorly elucidated. To address this, our manuscript aims to elucidate the intricate interplay between GD3S and WT or mutant p53 by employing a comprehensive strategy that analyzes data from BC patients and cell lines through various experimental approaches, aiming to shed light on the mechanistic aspects of their cooperative effects and reveal novel therapeutic vulnerabilities for BC treatment.

## Materials and methods

### Methods

#### Sex as a biological variable

Our study exclusively examined female mice because the disease modeled is only relevant in females.

#### Breast cancer cell lines

A panel of TNBC cell lines was purchased from ATCC (Manassas, VA) and cultured at 37 °C in a 5% CO_2_ incubator less than 6 months after revival, per ATCC recommendations, in either Roswell Park Memorial Institute medium or Dulbecco’s modified Eagle’s medium containing 10% fetal bovine serum (Gibco) and 1% penicillin/streptomycin (Mediatech, Corning Inc., Manassas, VA). The non-TNBC cell lines MCF7 and ZR751 were a kind gift from Dr. Naoto Ueno’s laboratory at MD Anderson Cancer Center. The patient-derived xenograft–derived TNBC cell line HIM3 was a kind gift from Dr. Piwnica-Worms’s laboratory at MD Anderson.

#### Inducible p53 knockdown and GD3S overexpression

To achieve inducible p53 knockdown and GD3S overexpression, we utilized lentiviral-mediated short-hairpin RNA (shRNA) for the stable knockdown of mutant p53 in TNBC cell lines harboring specific GOF p53 mutations. Further details on the generation of inducible p53 knockdown and GD3S overexpression can be found in the supplementary methods.

#### Flow cytometry

Breast cancer cells were subjected to trypsin and washed once with phosphate-buffered saline (PBS). The cells were then incubated for 30 min with 4 μL/reaction of allophycocyanin-conjugated purified anti-human ganglioside GD2 antibody (Cat. #357302, BioLegend, San Diego, CA). For GD3 expression analysis, cells were stained with 3 μL/reaction of unconjugated anti-GD3 antibody (clone R24, Millipore Sigma, Burlington, MA) for 30 min on ice and then washed twice with PBS. For secondary staining, the cells were incubated for 30 min with 100 μL of diluted goat anti-mouse antibody conjugated to Alexa Fluor 647 (Cat. #A21235, Life Technologies, Carlsbad, CA). Staining with 4′, 6-diamino-2-phenylindole (DAPI; Cat. #D1306, Thermo Fisher Scientific, Waltham, MA) was used to exclude dead cells. Allophycocyanin-conjugated purified mouse IgG2A antibody (Cat. #400219, BioLegend) was used as an isotype control to account for nonspecific antibody binding. After incubation, the cells were washed once with PBS containing 0.5 μg/mL DAPI and analyzed with an LSR II (BD Biosciences, Franklin Lakes, NJ) or Gallios (Beckman Coulter, Brea, CA) flow cytometer. Ten thousand events were acquired for each sample. All experiments were performed in duplicate, and all flow cytometry data were analyzed with FlowJo software (version 10, Ashland, OR).

#### Western blotting

Breast cancer cells (3 × 10^6^) were subjected to lysis. Proteins were harvested from the cell lysates using Laemmli buffer containing 50 μL/mL β-mercaptoethanol. Protease and phosphatase inhibitor cocktail was added to the protein lysates at a 1:1 ratio, and 15 μL of each protein sample was loaded onto 4–15% Mini-PROTEAN TGX precast gels for separation (Cat. #4561086, Bio-Rad Laboratories, Hercules, CA). Additional details are available in the supplementary methods.

#### Immunohistochemistry

We obtained formalin-fixed, paraffin-embedded (FFPE) archived primary breast tumor tissues expressing wild-type or mutant p53 from MD Anderson’s Tissue Bank, approved by MD Anderson’s Institutional Review Board (protocol #PA19-0732). Informed consent was obtained from all participants. Immunohistochemical (IHC) analysis of GD3S and p53 expression were conducted using standard methods. Additional details are available in the supplementary methods.

#### Live cell analysis for apoptosis assays

To assess the impact of doxycycline-induced mutant p53 knockdown on apoptosis, different Hs578T and BT549 clones (SCR_shp53-GD3S_EV, SCR_shp53-GD3S_OE, shp53-GD3S_EV, and shp53-GD3S_OE) were plated in 96-well plates (Corning) with 100 µL of growth medium and incubated for 12 h. The cells were then treated with vehicle or 1 µM doxycycline, and apoptosis was assessed by measuring Annexin V (Cat. #4642, Sartorius) binding and Caspase-3/7 (Cat. #4440, Sartorius) activity using the IncuCyte live-cell analysis system (Sartorius, Göttingen, Germany). Fluorescence signals were monitored at 1-h intervals for 12–24 h. Similarly, MCF7 and ZR751 cells expressing either an empty vector (EV) or GD3S overexpression (OE) were plated in 96-well plates and treated with increasing concentrations of Nutlin-3a (1.25–10 µM). The effect of WT p53 stabilization on apoptosis was measured by assessing Annexin V binding and Caspase-3/7 activity using the IncuCyte system.

#### Tumor spheroid assay

To assess the impact of doxycycline-induced p53 knockdown on the tumor spheroid–forming ability of different Hs578T and BT549 clones, we seeded SCR_shp53-GD3S_EV, SCR_shp53-GD3S_OE, shp53-GD3S_EV, and shp53-GD3S_OE cells in ultra-low attachment 96-well plates (Corning) at a density of 10 × 10^3^ cells in 100 µL of growth medium. After 3 days, the cells were exposed to either vehicle or 1 µM doxycycline, and spheroid formation and growth were assessed by using the IncuCyte live cell analysis system to monitor the brightfield area at 6-h intervals for 48–72 h.

#### Total RNA isolation and gene expression by real-time PCR

RNA extraction was performed using a RNeasy Mini Kit (Qiagen, Hilden, Germany) according to the manufacturer’s instructions. Further details can be found in supplementary method.

#### Chromatin immunoprecipitation qPCR

The GD3S promoter binding of p53 was assessed by chromatin immunoprecipitation (ChIP)-qPCR with 5 × 10^6^ cells per reaction. Further details on the ChIP-qPCR can be found in the supplementary methods.

#### Luciferase reporter gene assays

For the assessment of GD3S promoter activity, the *ST8SIA1* promoter (human) sequence spanning 2278 bp (2042 bp upstream and 236 bp downstream of the transcription start site) was cloned into the lentivirus promoter reporter plasmid pLL-CMV-Luciferase-T2A-Puro (Cat. #LL150PA/VA-1, System Biosciences, Palo Alto, CA) through the SpeI and NheI restriction enzyme sites and verified with Sanger sequencing. Further details on Luciferase assay can be found in the supplementary methods.

#### RNA sequencing

Stable MCF7 cells with or without GD3S overexpression were cultured in triplicate, treated with 10 µM nutlin-3a for 72 h, and then washed in PBS. RNA extraction was then performed using an RNA extraction kit (Qiagen) according to the manufacturer’s instructions. The extracted RNA was sent to LC Sciences (Houston, TX) for RNA-sequencing analysis. Additional information regarding the RNA-sequencing analysis is provided in the supplementary methods.

#### Tumor xenografts

All animal experiments were approved by MD Anderson’s Institutional Animal Care and Use Committee (Protocol # 1972-RN01) and were performed in accordance with all state and federal rules and regulations. 6–8 week-old female NSG mice were procured from Jackson Lab, Bar Harbor, ME (NOD.Cg-Prkdc Il2rg/SzJ). Additional details are available in the supplementary methods.

#### Data acquisition and statistical analysis

The characteristics of the patients whose archived FFPE tissue samples were used were extracted from MD Anderson’s electronic health records system (Epic Systems, Verona, WI). *ST8SIA1* and p53 expression was evaluated in 79 breast tumor samples. Histochemical scores (H-scores; calculated by multiplying the staining intensity by the percentage of positive cells) for GD3S and p53 expression were reported as means ± standard deviations. Pearson correlation coefficient (*r*) analysis was used to assess the correlation between GD3S and p53 expression. An independent *t*-test was used to compare the mean for binary categorical variables. One-way analysis of variance was used to compare the mean across categorical variables with more than 2 groups. *P* values less than 0.05 were considered significant. All analyses of patient and cell line data were performed using IBM SPSS software for Windows (version 26) and Prism software (version 9, GraphPad Software, La Jolla, CA). Figures were generated using the Prism software. All data generated or analyzed during this study are included in this published article and its supplementary information files.

## Results

### GD3S is differentially expressed in breast cancer with TP53 mutations

**Fig. 1:**
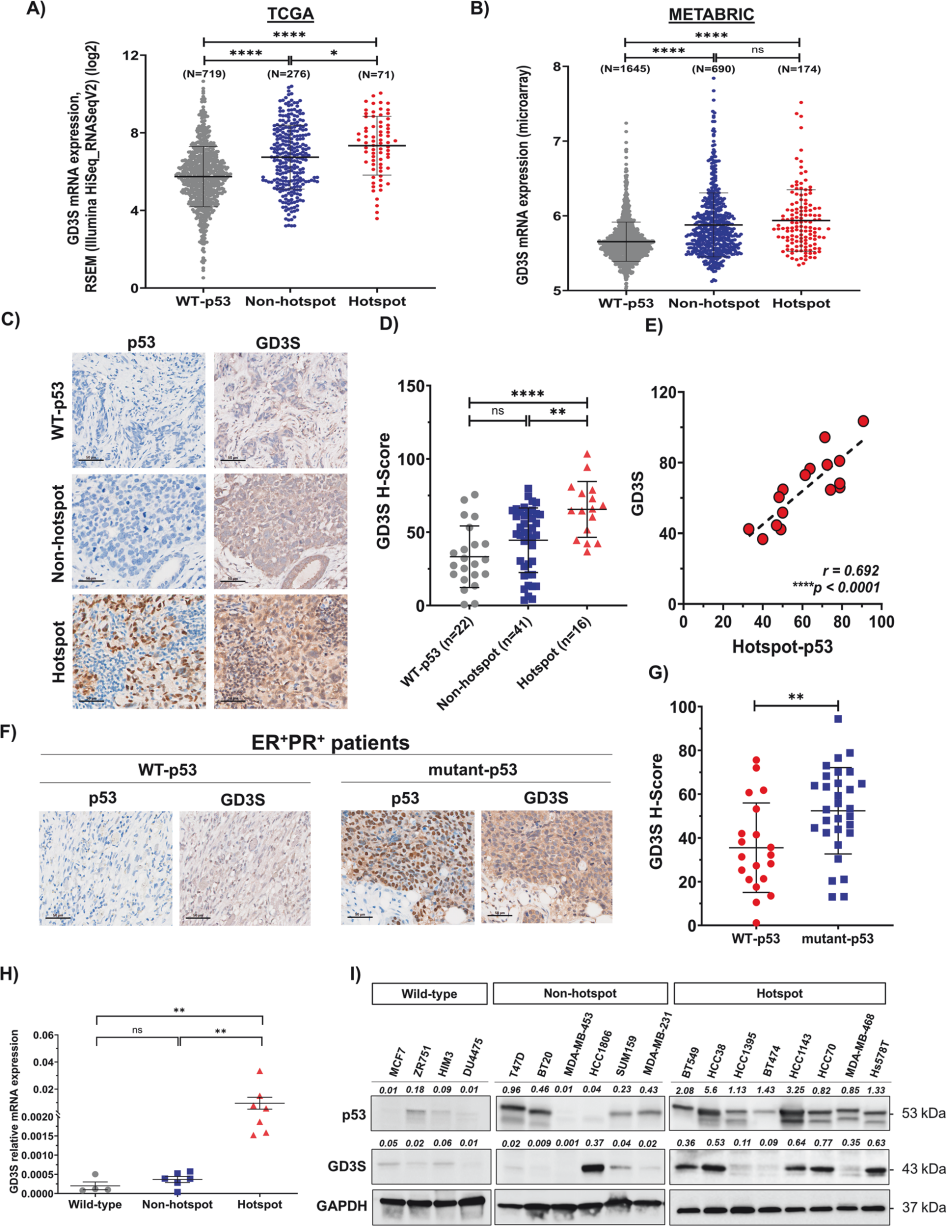
GD3S is overexpressed in breast cancer patients with p53 hotspot mutations. **A** In the TCGA and METABRIC breast cancer (BC) patient datasets, GD3S mRNA expression was correlated with p53 mutation status. In the TCGA dataset, GD3S mRNA expression was significantly higher in patients with p53 hotspot mutations (*n* = 71) than in patients with WT p53 (*n* = 719) or non-hotspot p53 mutations (*n* = 276). *P < 0.05; ****P < 0.0001. RSEM, RNA-seq by expectation-maximization. **B** Similarly, in the METABRIC dataset, GD3S expression was significantly higher in patients with non-hotspot (*n* = 690) or hotspot (*n* = 174) p53 mutations than in patients with WT p53 (*n* = 1645). ****P < 0.0001. **C** Representative IHC images of p53 and GD3S expression in archived FFPE samples from BC patients with WT p53 (*n* = 22), non-hotspot p53 mutations (*n* = 41), and hotspot p53 mutations (*n* = 16). **D** H-scores for GD3S expression in BC patients with different p53 expression status. **P = 0.006; ****P < 0.0001; ns, not significant. **E** Pearson correlation coefficient analysis revealed linear correlations between GD3S and p53 protein expression in patients with hotspot p53 mutations (*r* = 0.692). ****P < 0.0001. **F** Representative IHC images of p53 and GD3S expression in archived patient samples of ER^+^PR^+^ BC tissue with WT p53 (*n* = 20) or mutant p53 (*n* = 30). **G** H-scores for GD3S expression in the patient samples in (**F**). **P = 0.005. **H** Relative mRNA expression of GD3S in different BC cell lines with different p53 expression status. GD3S expression was significantly higher in cell lines with hotspot p53 mutations (*n* = 7) than in cell lines with WT p53 (*n* = 4) or non-hotspot p53 mutations (*n* = 6). **P = 0.02. **I** Western blotting for p53 and GD3S in breast cancer cell lines with WT p53 (*n* = 4), non-hotspot p53 mutations (*n* = 6), or hotspot p53 mutations (*n* = 8). GAPDH was used as a loading control. Throughout the figures, scale bar in IHC images represents 50 µm, and bar graphs show means and standard deviations.

To confirm and characterize in detail the association between GD3S expression and p53 mutation status in primary breast tumors, we analyzed more than 1000 patients in The Cancer Genome Atlas (TCGA) PanCancer and Molecular Taxonomy of Breast Cancer International Consortium (METABRIC) datasets [16]. Compared with those with WT p53, BC patients with p53 mutations had significant upregulation of GD3S expression (*P* < 0.0001) (Fig. 1A, B). In the TCGA dataset, GD3S expression was significantly higher in the presence of hotspot p53 mutations compared to non-hotspot mutations (*P* < 0.05) or WT p53 (*P* < 0.0001) (Fig. 1A). However, no significant differences in GD3S expression were observed between patients with different hotspot mutations (Figs. S1A and S1B). To validate these findings, we measured GD3S and p53 protein expression in archived FFPE primary tumor tissue samples (*n* = 79) with or without p53 mutations by IHC analysis. Of the 79 tumor samples analyzed, 22 (27.85%) had WT p53, 41 (51.9%) had non-hotspot p53 mutations, and 16 (20.25%) had hotspot p53 mutations. Consistent with our findings in the TCGA and METABRIC datasets, the expression levels of GD3S and p53 varied according to p53 mutation status among BC patient samples (Figs. 1C and S1C). Patients with WT p53 had low or negligible staining for GD3S and p53 (Figs. 1C, D and S1C). In contrast, patients with hotspot p53 mutations showed the highest GD3S expression (H-scores 65.56 ± 19.04) compared to patients with WT p53 (*P* < 0.0001) or non-hotspot TP53 mutations (*P* = 0.006) (Fig. 1D). Moreover, we found a significant positive correlation between p53 and GD3S expression in patients with hotspot p53 mutations (*r* = 0.692; *P* < 0.0001), but not in patients with non-hotspot p53 mutations (*r* = 0.04; *P* = 0.18) or WT p53 (*r* = 0.005; *P* = 0.76) (Figs. 1E and S1D, S1E).

Interestingly, we observed a similar phenomenon among a subset of estrogen and progesterone receptor–positive (ER^+^PR^+^) BC patients. Patients with p53 mutations (*n* = 30) in this subgroup had significantly elevated GD3S expression compared with those with WT p53 (n = 20; *P* = 0.005) (Fig. 1F, G). To further investigate the prognostic significance of GD3S in ER/PR^+^ breast cancer patients, we analyzed clinical parameters, including tumor grade, treatment details, and patient outcomes. Our analysis revealed that patients with p53 mutations spent a significantly shorter duration under treatment (29.03 ± 19.55 months) compared to those with WT p53 (73.95 ± 71.93 months, *P* < 0.05). This finding suggests that treatment was more effective in prolonging survival in WT p53 patients with lower GD3S expression, whereas patients with mutant p53 and high GD3S expression had poorer outcomes despite undergoing treatment (Table S1). In addition, among patients with p53 mutations, there was no significant difference in GD3S expression (*P* = 0.9) between ER^+^PR^+^ and TNBC patients (Fig. S1F and S1G). Together, these findings imply that GD3S expression in BC patients exhibits heterogeneity and is associated with p53 mutation status regardless of hormone receptor expression.

Next, to interrogate the effect of p53 mutations on the GD2 biosynthesis pathway, we first used the TP53 Cell Line Compendium to classify 24 available BC cell lines according to p53 mutation status (Table S2). Reverse phase protein array (RPPA) analysis of p53 expression levels from the MD Anderson Cell Lines Project (MCLP) revealed tightly regulated low p53 expression in cell lines with WT p53 (Fig. S2A). In contrast, cell lines with hotspot p53 mutations have the highest p53 expression levels and positively correlated with GD3S mRNA expression (Table S2, Figs. 1H and S2B). Moreover, flow-cytometry analysis unveiled differential expression of GD2^+^ and GD3^+^ cells among distinct BC cell lines characterized by different p53 mutation status (Table S2, Fig S2C and S2D). Compared to cell lines with WT p53, cell lines with p53 hotspot mutations had a significantly higher percentage of GD2^+^ cells (25.9% vs 3.4%; *P* = 0.02) as well as higher GD3S mRNA expression (*P* = 0.02). However, this was not observed with GD2 synthase (GD2S/*B4GALNT1*), indicating a strong correlation between GD3S expression and p53 mutation status (Figs. 1H and S2E).

To further validate the association between p53 mutation status and GD3S expression, we performed Western blotting to measure GD3S and p53 protein expression in 18 BC cell lines. In most cell lines with hotspot p53 mutations, both GD3S and p53 protein levels were upregulated, displaying a significant positive correlation (*r* = 0.85; *P* = 0.006) (Figs. 1I and S2F). In contrast, in cell lines with WT p53, both GD3S and p53 levels were reduced, showing a negative correlation (*r* = −0.12; *P* = 0.44). Conversely, a few cell lines with non-hotspot p53 mutations (T47D, BT20, SUM159, and MDA-MB-231) had elevated GD3S protein levels but very low p53 protein levels, resulting in a negative correlation, albeit a nonsignificant one (*r* = −0.43; *P* = 0.39). Taken together, these results indicate that GD3S expression in BC cells is strongly associated with specific p53 hotspot mutation status in both ER^+^ and TNBC subtypes.

### WT p53 downregulates GD3S expression

**Fig. 2:**
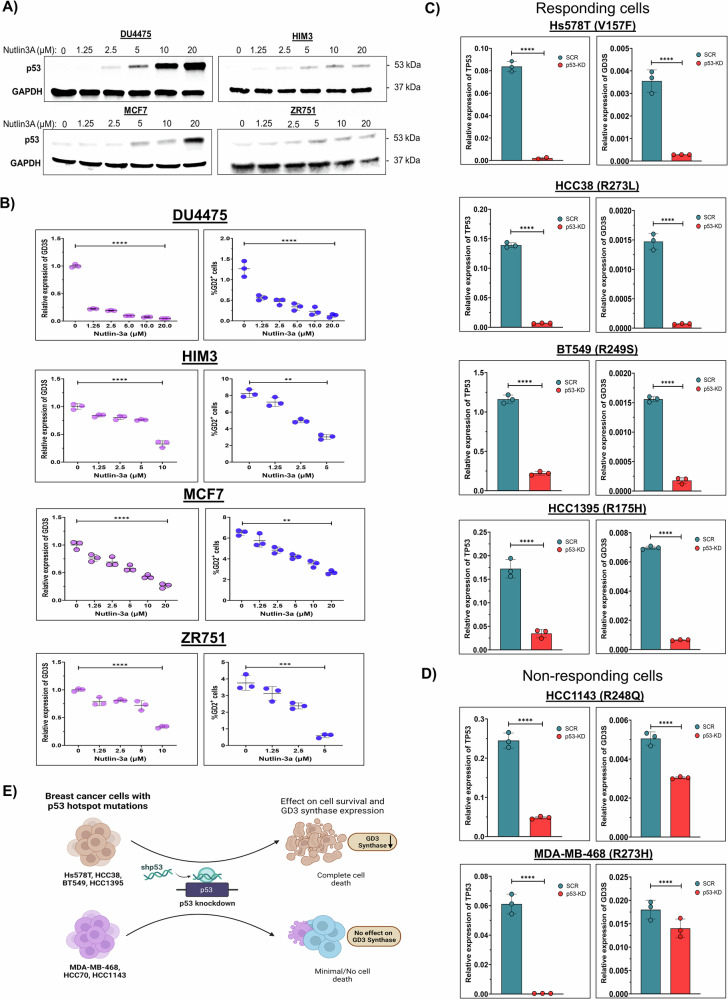
GD3S expression is inhibited in breast cancer cells with stabilized WT p53 and depleted mutant p53. **A** Western blotting for p53 and GAPDH (loading control) in DU4475, HIM3, MCF7, and ZR751 cells treated with different concentrations of nutlin-3a (0, 1.25, 2.5, 5, 10, or 20 µM) for 72 h. **B** Dose-dependent decreases in the relative mRNA expression levels of GD3S and the percentages of GD2-expressing cells were observed in HIM3, MCF7, and ZR751 cells treated with different concentrations of N3a for 72 h. **P < 0.01; ***P < 0.001; ****P < 0.0001. **C** In BC cell lines that responded to N3a (Hs578T, HCC38, BT549, and HCC1395) and have different p53 GOF mutations (V157F, R273L, R249S, and R175H, respectively), shRNA-mediated p53 knockdown significantly decreased GD3S (*ST8SIA1*) mRNA expression. ****P < 0.0001. **D** In BC cell lines that did not respond to N3a (HCC1143 and MDA-MB-468, with the p53 hotspot mutations R248Q and R273H, respectively) p53 knockdown only mildly decreased GD3S mRNA expression. ***P < 0.001; ****P < 0.0001. **E** Schematic diagram showing that p53 reduction significantly decreases GD3S expression and results in complete cell death in responding BC cells but has no or minimal effect on GD3S expression and cell death in non-responding BC cells.

Since we found a substantial relationship between the presence of p53 mutations and the expression of GD3S and GD2 in BC cells, we sought to elucidate the regulatory role of WT p53 in GD3S expression. To achieve this, we used the MDM2 inhibitor nutlin-3a (N3a) to stabilize p53 expression [17, 18] in BC cell lines with WT p53 (DU4475, HIM3, ZR751, and MCF7). N3a treatment increased p53 protein expression in a dose-dependent manner (Fig. 2A). Interestingly, treatment with N3a also led to a dose-dependent decrease in GD3S mRNA expression and reduction in the percentage of GD2^+^ cells in all four cell lines (Fig. 2B). Compared with their untreated counterparts, DU4475 and HIM3 cells treated with N3a (up to 20 µM) had 21-fold and 3-fold reductions in GD3S expression, respectively, and MCF7 and ZR751 cells had 3.5-fold and 3-fold reductions in GD3S expression, respectively (*P* < 0.05) (Fig. 2B). These results suggest that stabilization of WT p53 exerts a dominant influence leading to GD3S downregulation and a subsequent reduction in the GD2^+^ BC stem cell population.

### p53 GOF mutations confer a distinct survival advantage in breast cancer cells

**Fig. 3:**
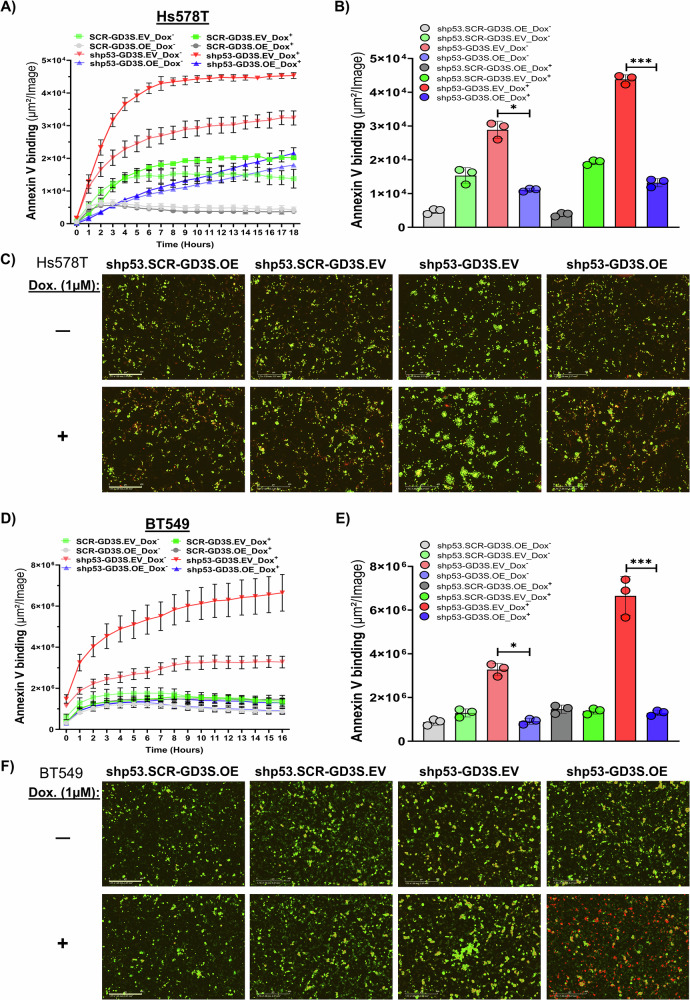
Increased GD3S expression counteracts the effect of p53 knockdown on the death of breast cancer cells with GOF p53 mutations. **A–F** Hs578T and BT549 cells expressing inducible p53-knockdown system were further genetically modified to stably overexpress GD3S and an empty vector (EV) control plasmid. Subsequently, these cells were treated with doxycycline (Dox;1 µM) to induce p53 knockdown. IncuCyte analysis of Hs578T cells (**A**, **C**) and BT549 cells (**D**, **F**) after p53 knockdown revealed that those without GDS3 overexpression had increased annexin V fluorescence compared with those overexpressing GD3S. Annexin V fluorescence levels were quantified using IncuCyte data analysis software. The representative images depict green pseudo-color for annexin v green staining, and the scale bar in IncuCyte images represents 400 µm. *P < 0.05; ***P < 0.001.

Next, to investigate the effect of mutant p53 on GD3S expression, we knocked down p53 in BC cell lines with different p53 mutations (Hs578T, HCC38, BT549, HCC1395, HCC1143, HCC70, and MDA-MB-468) by transient overexpression of shRNA targeting p53 mRNA (Fig. S4A). Interestingly, three of the cell lines (Hs578T, HCC38, and BT549) did not survive p53 knockdown (Table S3), and we observed growth arrest in another cell line (HCC1395), indicating that these cell lines depend on mutant p53 for their survival. In addition, GD3S mRNA expression significantly decreased upon p53 knockdown in those four cell lines (*P* < 0.0001) (Figs. 2C and S3A, S3B), which suggests that these GOF p53 mutations (V157F, R273L, R249S, and R175H) have regulatory roles in modulating transcription of GD3S. In contrast, p53 knockdown did not impact proliferation or survival of MDA-MB-468, HCC1143, and HCC70 cells. Moreover, expression of GD3S was also not altered upon p53 knockdown in these cells (Figs. 2D and 3C), indicating that p53 hotspot mutants R273H in MDA-MB-468 cells and R248Q in HCC1143 and HCC70 cells may not directly regulate GD3S expression (Fig. 2E).

Previous studies have shown that mutant p53 exerts a dominant negative effect by interfering with the functions of the WT p53 in human cancers [19–22]. To investigate the influence of mutant p53 on WT p53, we induced the ectopic expression of mutant p53 (R175H and R249S) in MCF7 and ZR751 cells. To stabilize WT p53, we treated the same cells with N3a. We found that mutant p53 upregulated GD2 levels and GD3S expression (Fig. S4A–S4C). However, upon dose-dependent stabilization of WT p53 with N3a, GD2 levels and GD3S expression were significantly reduced, even in the presence of ectopically expressed mutant p53 protein. This indicates that the stabilization of WT p53 predominantly suppresses the effects of mutant p53 (*P* < *0.01*, *P* < 0.001, *P* < 0.0001). Next, to investigate the influence of WT p53 on mutant p53, we induced the transient expression of WT p53 in BT549, Hs578T, and HCC1395 cells. We found that exogenous expression of WT p53 in these BC cells with mutant p53 led to the downregulation of GD3S at both the mRNA and protein levels (*P* < 0.001, *P* < 0.0001; Fig. S4D, S4E). This observation challenges the established notion that mutant p53 exerts a dominant negative effect [19–22] and shows that WT p53 must be absent for the effects of mutant p53 to fully manifest.

### GD3S alone can inhibit p53-mediated apoptosis in breast cancer cells

To prevent mutant p53 knockdown–mediated cell death and validate the regulation of GD3S expression by p53, we generated stable doxycycline-inducible p53 shRNA–expressing versions of cell lines Hs578T (V157F) and BT549 (R249S) (Fig. S3A and S3B). These cells were further engineered to stably overexpress GD3S, enabling us to evaluate the effect of GD3S on mutant p53-dependent survival mechanisms in TNBC cell lines. To assess the role of GD3S in apoptosis resistance, we treated these inducible cell lines and their parental controls with or without doxycycline (1 µM) and monitored apoptotic responses via annexin V binding using the IncuCyte live-cell imaging system. As expected, p53 knockdown significantly increased annexin V fluorescence, indicating an apoptotic response in both Hs578T (V157F) and BT549 (R249S) cells. However, GD3S overexpression rescued cells from apoptosis, leading to a >70% reduction in annexin V–positive apoptotic cells compared to p53-knockdown EV controls (*P* < 0.001) (Fig. 3A–F). To further elucidate the mechanism of GD3S-mediated apoptosis inhibition, we analyzed caspase-3/7 activity, which represents executioner caspases and serves as a key indicator of late-stage apoptosis involving the intrinsic mitochondrial apoptotic pathway [23, 24]. Live-cell imaging revealed that GD3S overexpression significantly suppressed caspase-3/7 activation in p53-depleted Hs578T and BT549 cells, reinforcing its role in apoptotic resistance (*P* < 0.0001, Fig. S5A and S5B). Since caspase-3/7 activation requires mitochondrial outer membrane permeabilization and cytochrome c release, these findings suggest that GD3S inhibits both early (annexin V) and late (caspase-3/7) apoptosis via mitochondrial stabilization. Overall, these findings support a novel anti-apoptotic role of GD3S.

**Fig. 4:**
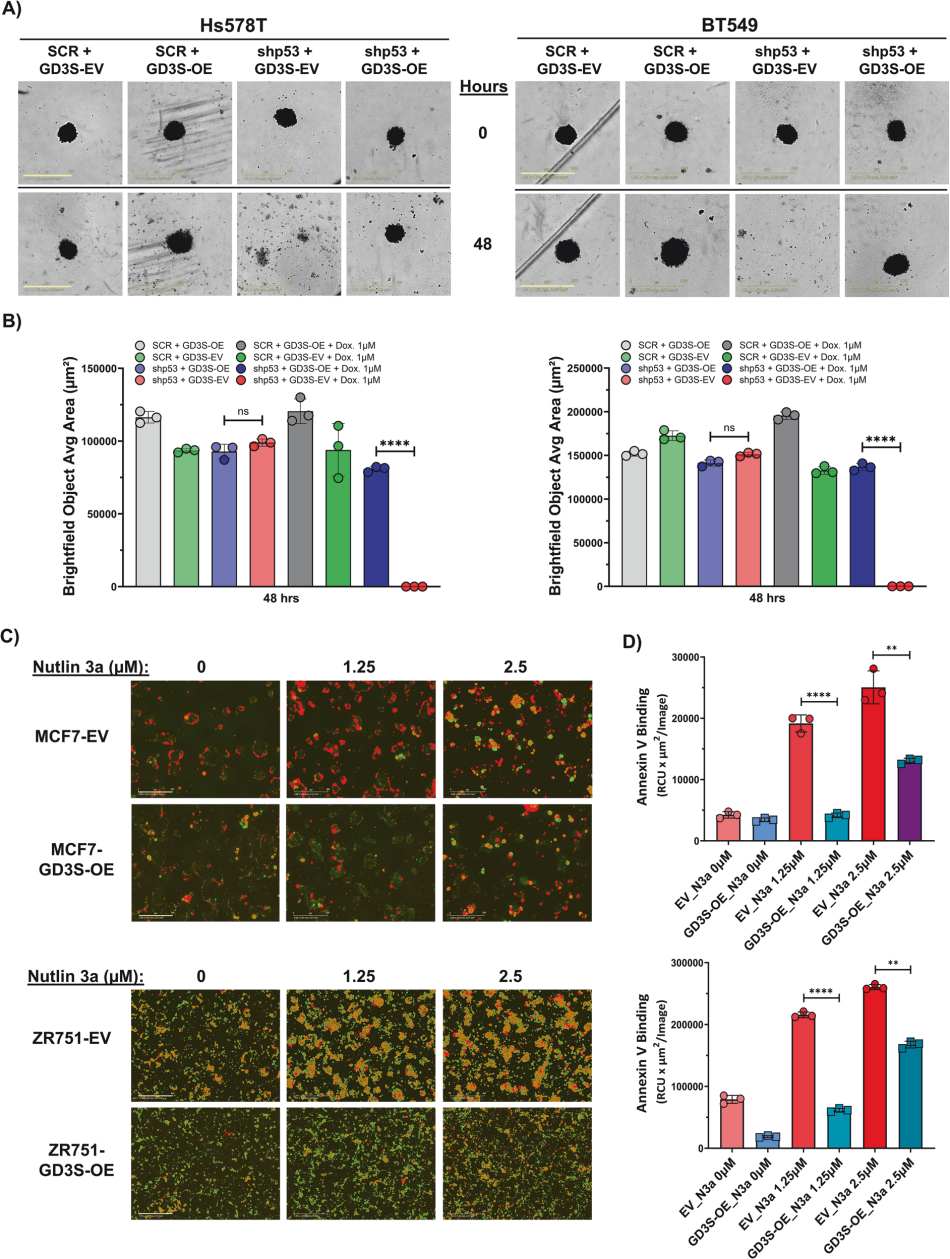
Elevated GD3S expression provides a survival advantage to breast cancer cells that have mutant or WT p53 and typically undergo p53-mediated cell death. **A**, **B** The effects of GD3S overexpression on the tumor spheroid–forming ability of Hs578T and BT549 cells were assessed after doxycycline (Dox) treatment to induce p53 knockdown. Representative IncuCyte brightfield images of tumor spheroids arising from Hs578T and BT549 clones with inducible p53 knockdown and with or without GD3S overexpression at 0 and 48 h are shown (Scale bar: Hs578T–800 µm, BT549–900 µm). The brightfield object areas for spheroids were quantified using IncuCyte data analysis software (**B**). **C** The effect of N3a (0, 1.25, or 2.5 µM) on the apoptosis of MCF7 and ZR751 cells with or without GD3S overexpression was assessed with annexin V binding using the IncuCyte system (Scale bar: MCF7–200 µm; ZR751–400 µm). **D** The annexin V fluorescence levels were quantified using IncuCyte data analysis software. The representative images depict red pseudo-color for annexin v red staining. ****P < 0.0001; **P < 0.01.

We endeavored to validate these findings using a xenograft mouse model by orthotopically implanting different clones of Hs578T and BT549 into the mammary fat pads of NSG mice. Given the inability of these cells to form tumors in vivo, we focused on evaluating the impact of GD3S on rescuing the tumor-promoting properties and clonogenic potential of these clones following p53 knockdown. We performed 3-dimensional (3D) tumor spheroid assays, mammosphere assays, and 2-dimensional (2D) colony-formation assays. After treatment with doxycycline to induce p53 knockdown, tumor spheroids comprising Hs578T and BT549 cells without GD3S overexpression completely disintegrated within 48 h, whereas those comprising cells with GD3S overexpression did not (Fig. 4A, B). Even with p53 knockdown, the mammosphere-forming and colony-forming abilities of GD3S-overexpressing Hs578T and BT549 cells showed no significant changes compared to cells without GD3S overexpression (Fig. S6A and S6B). These findings offer compelling evidence that GD3S acts as a suppressor of apoptosis in TNBC cells with specific GOF p53 mutations.

Next, to investigate the impact of GD3S on the apoptosis of ER^+^PR^+^ BC cells with WT p53, we subjected stable MCF7 and ZR751 cell lines with or without GD3S overexpression to different doses of N3a (0–2.5 µM). The apoptotic response following p53 stabilization was assessed via annexin V binding using the IncuCyte system. Apoptosis induced by p53 stabilization with N3a was significantly increased in a dose-dependent manner (*P* < 0.0001, *P* < 0.01) (Fig. 4C, D). Conversely, the apoptotic effect was diminished in GD3S-overexpressing cells, suggesting that GD3S helps protect cells against apoptosis induced by WT p53. Consistent with these findings, Caspase-3/7 IncuCyte analysis in MCF7 cells revealed a similar trend, where GD3S overexpression reduced Caspase-3/7 activation following N3a treatment (Fig. S5C). Together, these results establish a novel anti-apoptotic function of GD3S in BC cells, irrespective of p53 mutation status.

### WT p53 and p53 with GOF mutations exhibit distinctive transcriptional regulation of GD3S by directly binding to its promoter

**Fig. 5:**
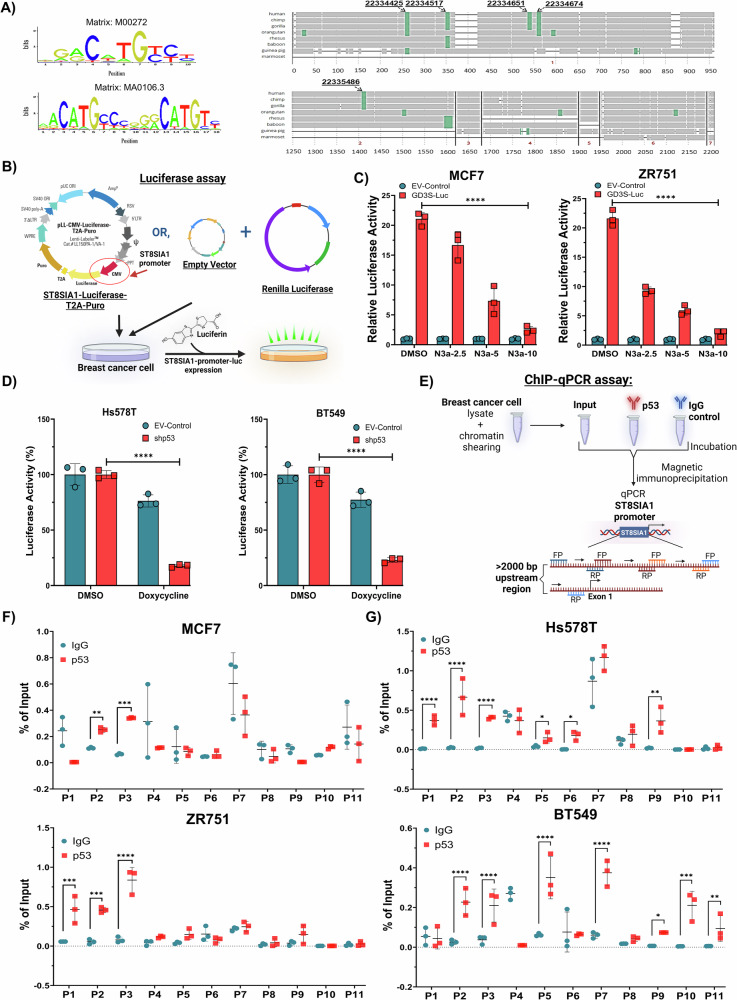
GD3S promoter activity is directly regulated by WT and mutant p53. **A** The ConTra v3 web server was used to identify consensus p53 binding sites on the upstream promoter regions and the conserved p53 binding regions in the more than 2000 nucleotides upstream of the GD3S promoter region across various species. **B** Schematic diagram illustrating the promoter-luciferase assay methodology. A luciferase reporter system was used to measure the luciferase activity directly regulated by the GD3S promoter after the cloning of more than 2000 nucleotides in the 5’ upstream promoter region of the GD3S gene. Luciferase activity was visualized through the transient expression of GD3S promoter-luciferase, EV-luciferase, and Renilla luciferase plasmids in BC cells. **C** MCF7 and ZR751 cells were transfected with GD3S-promoter-luciferase and control plasmids and then treated with increasing concentrations of N3a (0, 2.5, 5, and 10 µM) to assess the effect of p53 stabilization on GD3S promoter activity. Relative luciferase activity was measured after the luminescence of GD3S-promoter-luciferase was normalized to that of Renilla luciferase as the internal control. ****P < 0.0001. **D** Hs578T and BT549 cells with inducible p53 knockdown were transfected with GD3S-promoter-luciferase and control plasmids. The cells were then treated with doxycycline (1 µM) to induce p53 knockdown, and the effect of p53 knockdown on GD3S promoter activity was assessed. The luminescence of GD3S-promoter-luciferase was normalized to that of Renilla luciferase, and the fold decrease in luciferase activity was determined. ****P < 0.0001. **E** Schematic diagram illustrating the ChIP-qPCR assay methodology. FP, forward primer; RP, reverse primer. **F** ChIP-qPCR analysis showed a higher percentage of p53 binding enrichment on the specific GD3S upstream promoter region in MCF7 and ZR751 cells treated with an anti-p53 antibody than in those treated with the control non-immune IgG antibody. **P < 0.01; ***P < 0.001; ****P < 0.0001. **G** ChI*P*-qPCR analysis revealed a higher percentage of p53 binding enrichment on various regions of the GD3S upstream promoter in Hs578T and BT549 cells treated with an anti-p53 antibody than in those treated with the control non-immune IgG antibody. *P < 0.05; **P < 0.01; ***P < 0.001; ****P < 0.0001.

Analysis of the GD3S promoter using the ConTra v3 web server (Ghent University, Belgium) revealed the presence of 5 conserved putative p53 binding sites in the >2.0-kilobase (kb) region across humans, chimpanzees, and gorillas (Fig. 5A) [25]. To determine whether p53 regulates GD3S promoter for its transcription, we cloned a more than 2.0-kb region of the GD3S promoter, spanning 2042 base pairs (bp) upstream and 236 bp downstream of the transcription start site, into a promoter-luciferase reporter plasmid (Fig. 5B). The GD3S promoter-luciferase construct and an EV control plasmid, along with Renilla luciferase as an internal control for normalization, were transfected into MCF7 and ZR751 cells. We then evaluated the promoter-luciferase activity in the presence or absence of various concentrations of N3a (0–10 µM) and found that increasing levels of WT p53 resulted in a dose-dependent repression of GD3S promoter activity in both cell lines (*P* < 0.0001) (Fig. 5C). To investigate the effect of mutant p53 on GD3S promoter activity, we introduced the GD3S promoter-luciferase construct along with control plasmids into Hs578T and BT549 cells stably transfected with a doxycycline-inducible p53 shRNA construct. Compared with the vehicle control, doxycycline (1 µM) induced knockdown of p53 significantly suppressed GD3S promoter activity. The promoter activities in Hs578T and BT549 cells were reduced by more than 5- and 4-fold, respectively (*P* < 0.0001) (Fig. 5D).

Next, to determine direct binding of p53 to the GD3S promoter, we conducted a chromatin immunoprecipitation–quantitative PCR (ChIP-qPCR) assay. Magnetic IP was performed in cell lines with WT or mutant p53 to capture chromatin fragments bound to anti-p53 or immunoglobulin control antibody. The eluted chromatin was subjected to qPCR using the 11 primer pairs (Fig. 5E and Table S5). The ChIP-qPCR analysis demonstrated a notably increased percentage of input at the P1 and P2 primers in MCF7 cells and at the P1, P2, and P3 primers in ZR751 cells, signifying WT p53 enrichment at these specific regions of the GD3S upstream promoter in these cells (*P* < 0.01, *P* < 0.001, *P* < 0.0001) (Fig. 5F). However, the percentage of input was increased at the P1, P2, P3, P5, P6, and P9 primers in Hs578T cells and at the P2, P3, P5, P7, P9, P10, and P11 primers in BT549 cells, indicating distinct enrichment patterns of mutant p53 at the GD3S upstream promoter in cells with GOF p53 mutations (*P* < 0.05, *P* < 0.01, *P* < 0.001, *P* < 0.0001) (Fig. 5G). This highlights that both WT and mutant p53 can directly influence GD3S transcription by binding in distinct ways to its upstream promoter regions.

### GD3S overcomes WT p53–mediated tumor growth arrest in vivo

**Fig. 6:**
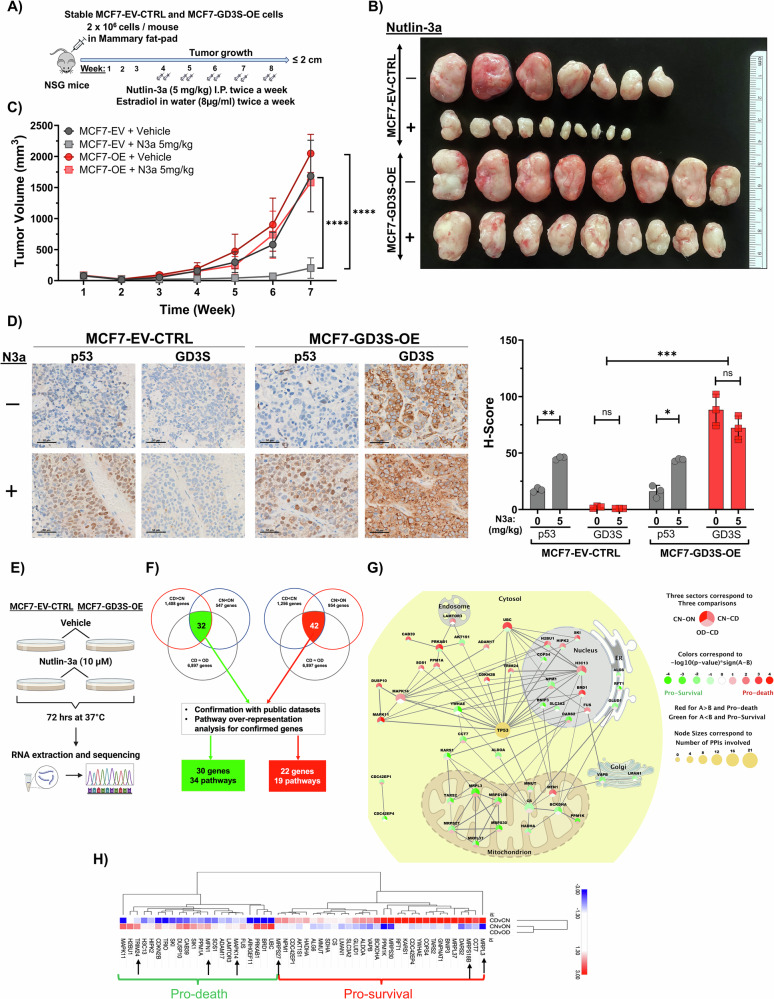
GD3S overexpression diminishes p53-mediated apoptosis in xenograft models by downregulating pro-death gene signature. **A** Schematic diagram illustrating the studies with MCF7 cell–derived xenografts with (MCF7-GD3S-OE) or without (MCF7-EV-CTRL) GD3S overexpression implanted into the mammary fat pads of female NSG mice (NOD.Cg-Prkdc Il2rg/SzJ; *n* = 10 per group), which were given estradiol in drinking water at a final concentration of 8 µg/mL. After tumors became palpable (~80 mm^3^), mice were randomized to receive N3a (5 mg/kg) or vehicle treatments. I.P. intraperitoneal. **B**, **C** Tumor volumes were measured twice weekly using calipers. When the diameters of their tumors were approximately 2 cm (~8 weeks after cell implantation), the mice were euthanized, and the tumors were imaged and weighed. ***P < 0.001. **D** Tumor samples were fixed in formalin, and IHC was used to assess p53 and GD3S expression, which was visualized with a Vectra Polaris microscope (Scale bar −50 µm) and quantified using Visiopharm image analysis software. ns = non-significant; *P < 0.05; **P < 0.01; ***P < 0.001. **E** Schematic diagram showing treatment conditions for RNA sequencing. **F** Venn diagram illustrates genes and related pathways after applying statistics and machine learning MATLAB toolbox for the enrichment. Conditions are labeled as follows: CD = control plasmid + DMSO, OD = GD3S-overexpression + DMSO, CN = control plasmid + N3a, ON = GD3S-overexpression + N3a. **G** Protein-protein interaction (PPI) network model connecting 52 of the 74 target genes. Each node represents a protein, and each node split into 3 sectors, representing log2 fold changes in all 3 categories with vs. without N3a treatments. Green represents pro-survival genes, red indicates pro-death genes for the top two sectors, and deeper colors indicate larger absolute values. **H** Heatmap displaying 52 genes categorized into pro-survival (*n* = 30) and pro-death (*n* = 22), with a black arrow indicating the chosen gene signature for validation through qPCR (refer to Fig. S7C and S7D).

To investigate the role of GD3S in preventing p53-mediated apoptosis and promoting tumor growth in vivo, we implanted MCF7 cells stably overexpressing GD3S (MCF7-GD3S-OE) or EV control (MCF7-EV-CTRL) plasmids (2 × 10^6^ cells/mouse) into the mammary fat pads of NSG mice (NOD.Cg-Prkdc Il2rg/SzJ; *n* = 10 per group) under estradiol treatment (Fig. 6A). Once tumors were established (100 mm^3^), the mice were randomized to tumor size–matched treatment groups and received intraperitoneal injections of either N3a (5 mg/kg) or vehicle twice weekly for 7 weeks. Despite the loss of some mice for unexplained reasons, we observed that N3a treatment significantly decreased tumor growth in mice implanted with MCF7-EV-CTRL cells. However, there was no effect of N3a treatment on tumor growth in mice implanted with GD3S-overexpressing MCF7 cells (*P* < 0.001) (Fig. 6B, C). IHC analysis for p53 and GD3S in the resected tumor samples confirmed that N3a treatment stabilized p53 in both the MCF7-EV-CTRL and MCF7-GD3S-OE mice groups (*P* < 0.05, *P* < 0.01). Notably, substantial GD3S expression was observed exclusively in the MCF7-GD3S-OE group, and there was no significant alteration in GD3S expression after N3a treatment in the GD3S-overexpressing group (Fig. 6D). These findings reinforce our in vitro observations and illustrate that GD3S effectively suppresses WT p53–mediated apoptosis and promotes tumor growth in vivo, demonstrating the pro-tumorigenic potential of GD3S.

### GD3S enhances mitochondrial activity to resist p53-induced cell death

Our investigation revealed that GD3S is a novel resistance factor in p53-mediated apoptosis in BC. To elucidate the downstream anti-apoptotic mechanism of GD3S, we treated MCF7 cells stably expressing the GD3S-overexpression plasmid or its EV-control plasmid with N3a, followed by RNA extraction and sequencing (Fig. 6E). After filtering for transcripts with average fragments per kilobase per million lower than 1.00, we identified 12,373 unique genes. Among these, utilizing the statistics and machine learning toolbox, we further identified 74 genes that were differentially expressed between each pair of conditions (including conditions with and without GD3S overexpression and with and without N3a treatment). These were subsequently refined by the identification of 52 genes associated with mitochondrial activity and cell death, highlighting a panel of 30 pro-survival and 22 pro-death genes (Fig. 6F). Moreover, protein-protein interaction network analysis using STRING pathway analysis revealed interactions of these 52 genes across diverse subcellular compartments (Fig. 6G). A ConsensusPathDB 5 (Release 35) analysis of the 52 genes revealed 34 pathways overrepresented (*P* < 0.01) by the 30 pro-survival genes and 19 pathways overrepresented by the 22 pro-death genes (Figs. 6H and S7A, S7B). Validation of RNA-sequencing data through qPCR for selected genes demonstrated significantly decreased expression of pro-death genes *MFN1*, *MAPK14*, and *TRIM24* in N3a-treated GD3S-overexpressing (MCF7-OE-N3a) cells compared to controls (MCF7-EV-N3a) (*P* < 0.001; Fig. S7C). Conversely, we observed upregulation of pro-survival genes *MRPL3*, *MRPS27*, and *MRPS18B* in GD3S-overexpressing MCF7 cells (*P* < 0.001; Fig. S7D). These results highlight the critical role of GD3S in alleviating apoptosis and cell death mediated by WT p53.

**Fig. 7:**
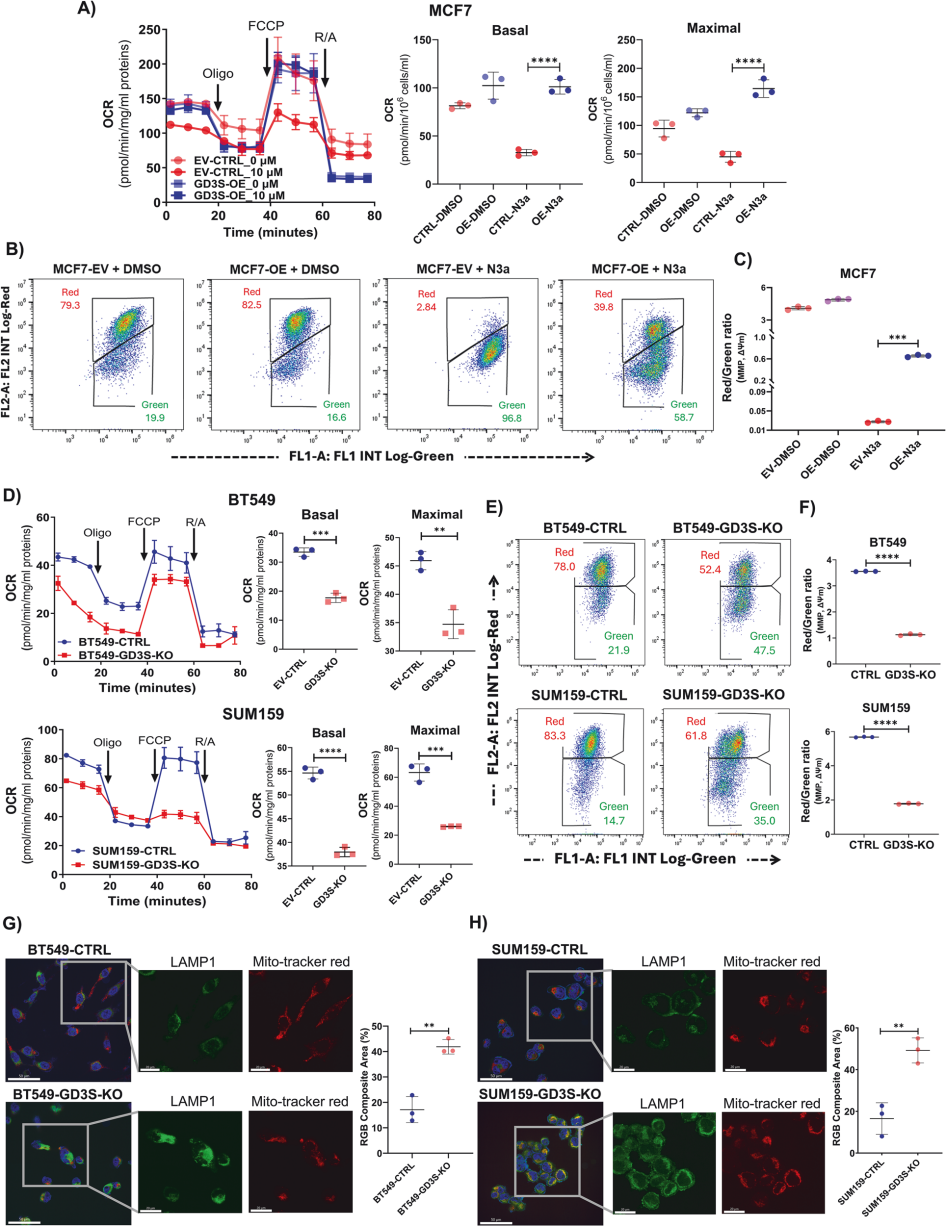
GD3S enhances mitochondrial activity to evade p53-induced apoptosis in breast cancer. **A** Seahorse assay indicating basal and maximal oxygen consumption rates (OCR) in MCF7 control and GD3S overexpressing cells after N3a treatment (10 µM). Oligo Oligomycin, FCCP Carbonyl cyanide-p-trifluoromethoxyphenylhydrazone, R/A Rotenone/Antimycin A. ****P < 0.0001. **B**, **C** The representative flow cytometry dot-blot image depicts the mitochondrial membrane potential (MMP; ΔΨm) in MCF7 cells with stable GD3S overexpression and control plasmid, stained with the JC-1 cationic dye, following treatment with vehicle (DMSO) and N3a (10 µM). The graph illustrates the ratio of the red J-aggregate to the green monomeric form of JC-1 treated with N3a (10 µM) for 48 h (**C**). ***P < 0.001 **D** Seahorse assay indicating basal and maximal oxygen consumption rates in BT549 and SUM159 cells after CRISPR-Cas9–mediated deletion of GD3S compared to their controls. *P < 0.05; **P < 0.01; ***P < 0.001. **E**, **F** The flow cytometry image displays a comparative assessment of MMP in BT549 and SUM159 cells with GD3S knockout and their control counterparts. The *x*-axis corresponds to the green fluorescence for the monomeric form of JC-1, while the *y*-axis represents the red fluorescence for the J-aggregate. MMP was examined in GD3S-knockout BT549 and SUM159 cells, along with their respective control cells (**F**). ****P < 0.0001. Confocal microscopy showing LAMP1-green and MitoTracker-red staining in BT549 (**G**) and SUM159 (**H**) GD3S-KO and their control cells. Scale bar: Main −50 µm, Inset −20 µm. **P < 0.01.

To determine the functional role of GD3S in WT p53–mediated apoptosis, we conducted a Seahorse assay to evaluate oxygen consumption rate (OCR) and mitochondrial stress. Notably, N3a-treated cells overexpressing GD3S exhibited enhanced mitochondrial respiration (basal and maximal) compared to those with the EV-control plasmid (*P* < 0.0001; Fig. 7A). The investigation into mitochondrial activity was further extended to the mitochondrial membrane potential (MMP; ΔΨm) using the JC-1 cationic dye. Consistent with our previous observations, GD3S overexpression in MCF7 cells led to an increase in MMP, counteracting the impact of WT p53 stabilization. This was evidenced by a significant shift from the green fluorescent monomeric form to the red fluorescent J-aggregate, indicating a markedly polarized mitochondrial state in MCF7-GD3S-OE cells compared to EV-control cells (*P* < 0.001; Fig. 7B, C). Next, we investigated whether GD3S loss alters mitochondrial function in BT549 and SUM159 cells, which harbor mutant p53. CRISPR-Cas9-mediated GD3S knockout significantly reduced OCR in BT549 and SUM159 cells, emphasizing the critical role of GD3S in mitochondrial dynamics (Fig. 7D). Consistently, the deletion of GD3S induced mitochondrial stress, as evidenced by a shift from the red fluorescent J-aggregate to the green fluorescent monomeric form, indicating decreased MMP in BT549 and SUM159 GD3S-knockout cells compared to controls (*P* < 0.0001; Fig. 7E, F).

To further examine whether GD3S loss–induced mitochondrial stress facilitates lysosome-mediated mitophagy, we quantified lysosomal and mitochondrial content, assessing autophagosome formation, a crucial step in mitophagy. Confocal microscopy confirmed that BT549 and SUM159 GD3S-knockout cells exhibited significantly increased lysosomal accumulation relative to mitochondria, compared to controls (*P* < 0.01; Fig. 7G, H).

Collectively, these findings suggest that GD3S inhibits p53-induced apoptosis and cell death through altering mitochondrial activity.

## Discussion

In this study, we demonstrate a previously unknown anti-apoptotic and tumor-promoting role of the sialyltransferase GD3 synthase, emphasizing its ability to circumvent the regulatory effects of both WT and mutant p53 in BC. Our findings reveal, for the first time, that WT p53 downregulates GD3S expression, whereas p53 with specific GOF mutations upregulates GD3S expression, thereby promoting a GD2^+^ BC stem cell phenotype and providing a survival advantage to BC cells. We sought to identify the mechanism behind this, and our results illustrate direct involvement of both WT and mutant p53 in the transcriptional regulation of GD3S. Moreover, they uncover that GD3S acts as a resistance factor in p53-mediated apoptosis by suppressing expression of pro-apoptotic genes and elevating expression of genes that enhance mitochondrial activity. These findings provide insight into the intricate relationship between p53 and GD3S, highlighting their significant impact on BC biology.

Mutations in p53 have been consistently linked to reduced survival rates among patients with different BC subtypes [26, 27]. Eighty percent of TNBC patients have p53 mutations, and these mutations demonstrate considerably more prognostic significance for TNBC patients than for non-TNBC (ER^+^PR^+^ BC) patients [28–31]. In the present study, contrary to expectations, 60% of ER^+^PR^+^ BC patients had p53 mutations with elevated p53 and GD3S levels. However, the expression levels of p53 and GD3S did not differ significantly between non-TNBC and TNBC patients with p53 mutations. These findings indicate that the increased expression of GD3S is directly associated with the presence of p53 mutations regardless of hormone receptor status.

Our findings suggest that four p53 mutations known for their alleged dominant negative effect and GOF activity, specifically R175H, R249S, V157F, and R273L, engage GD3S to exert their oncogenic effects [32–35]. Intriguingly, the introduction of WT p53 restrained the activity of these p53 mutants and simultaneously reduced the expression of GD3S. Converse of previous findings, this implies a dominant effect of WT p53 over mutant p53, aligning with a recent study emphasizing the significance of WT p53 and the absence of mutant p53’s dominant negative effect across various human tumors [36]. Additionally, a recent study noted no impact on cellular growth and proliferation upon deleting various p53 mutants in cell lines representing different tumor types. Notably, the introduction of WT p53, but not the deletion of mutant p53, suppressed tumor growth [37]. Our findings align with one aspect of this study, affirming the significance of WT p53, which must be absent for the effects of mutant p53 to fully manifest. However, unlike the aforementioned study, we observed that the survival of BC cells depends on the presence of specific p53 GOF mutations (R175H, R249S, V157F, and R273L). As cells with these mutations were unable to sustain the abrogation of mutant p53, we introduced a doxycycline-inducible p53 knockdown system to regulate p53 expression and conduct downstream analysis. Our in-depth analysis substantiated an essential role of GD3S as a regulator of tumor growth in BC with TP53 mutations. We have found that specific p53 mutations exert their GOF properties, including oncogenic activity, through GD3S. In contrast, Wang et al. primarily examined cell lines bearing non-hotspot p53 mutations and did not explore any downstream factors responsible for the GOF activity of hotspot p53 mutants in tumor growth [37]. Our observations find support in a lymphoma study highlighting the gene-selective neomorphic GOF activity of mutant p53 driving tumorigenesis [38]. Interestingly, a context-dependent response to the p53 mutation at position R273 was observed. Cells harboring the p53-R273H mutation displayed minimal effects on cell survival and GD3S expression in contrast to those with the p53-R273L variant. The p53-R273H mutation has been reported to manifest distinct phenotypic characteristics, along with reduced tumorigenicity compared to p53-R273C and p53-R273L mutations [39, 40].

The inhibitory effect of WT p53 on the GD3S promoter activity is in accordance with its recognized function of directly repressing genes linked to cancer stem cell characteristics, such as *CD44*, *NANOG*, and *PROM1* (CD133), through binding to their respective upstream promoters [41–43]. In contrast, mutant p53 with GOF activity has been shown to transcriptionally upregulate cancer stem cell–associated genes and genes associated with epithelial-to-mesenchymal transition, such as Snail and Zeb [10, 44]. Moreover, our previous findings highlight the involvement of the NF-κB pathway in regulating GD3S expression [45, 46]. Interestingly, WT p53 inhibits, while mutant p53 upregulates the NF-κB pathway, suggesting that the NF-κB pathway is likely a downstream modulator of *TP53* in regulating GD3S expression [47–49]. These findings reinforce our existing data and underscore the pivotal role of GD3S in mediating the contrasting effects of WT and mutant p53 on cancer stemness and epithelial-mesenchymal transition by regulating expression of gangliosides in BC [46]. Moreover, RNA-sequencing data validated that GD3S contributes to the suppression of several pro-apoptotic proteins, including *MFN1, MAPK14*, and *TRIM24* [50–52]. This suppression likely protects against p53-mediated mitochondrial apoptosis. Interestingly, GD3S also promotes the expression of several mitochondrial ribosomal genes, such as *MRPL3, MRPS27*, and *MRPS18B*, implying that GD3S may enhance mitochondrial protein synthesis and biogenesis, potentially strengthening mitochondrial activity and further contributing to BC survival [53]. Our functional validation assays substantiated that GD3S directly enhances mitochondrial activity. We observed a significant elevation in OCR and MMP (ΔΨm) with increased GD3S expression, countering the pro-apoptotic effects of WT p53 [54]. Conversely, upon genetic ablation of GD3S, we noted a substantial reduction in OCR and MMP, along with an enhanced tendency towards mitophagy. Consistently, our live-cell IncuCyte analysis demonstrated that Caspase-3/7 activation, a key marker of intrinsic mitochondrial apoptosis, was significantly suppressed in GD3S-overexpressing cells following p53 stabilization, further reinforcing its role in apoptotic resistance [23, 24]. This underscores the exclusive role of GD3S in sustaining mitochondrial activity amidst cellular stress.

The role we have uncovered for GD3S could act through known pathways downstream of GD3S. For instance, we previously demonstrated that the nutrient-sensing mammalian target of rapamycin (mTOR) pathway is upregulated in GD2^+^ BC cells, and inhibition of GD2 expression through GD3S blockade leads to downregulation of mTOR signaling [6, 7]. mTOR signaling enhances mitochondrial biogenesis and energy production, supporting cancer cell growth and survival [55–58]. In contrast, WT p53 inhibits the mTOR pathway, reducing mitochondrial function and promoting apoptosis [59, 60]. Additionally, Furukawa’s group has also shown that gangliosides downstream of GD3S, including GD3 and GD2, are involved in activation of FAK signaling [61]. These findings suggest that GD3S exerts its anti-apoptotic and mitochondrial activity through the FAK-mTOR signaling pathway [58]. Cancer cells often evade cell death by amplifying the expression of anti-apoptotic proteins, such as B-cell lymphoma 2 (BCL2) [62, 63]. Earlier, we have reported that knockdown of GD3S dramatically inhibited expression of BCL11A, a transcription factor playing a pivotal role in promoting BC stem cell activity and resistance to apoptosis [7, 64, 65]. These data provide another mechanistic link for GD3 as an anti-apoptotic protein in BC cells.

Our findings not only underscore the potential significance of GD3S as a promising prognostic biomarker in BC but also elucidate its implications in tumor aggressiveness and disease prognosis. The differential expression of GD3S, contingent upon the presence of p53 mutations, suggests its potential utility as a molecular marker for identifying patients with high-risk tumors and poor prognoses. Moreover, the positive correlation between p53 and GD3S expression in BC with hotspot p53 mutations identifies a potential source of the resistance of these cancer cells to therapeutic intervention, possibly stemming from the augmented mitochondrial activity and subsequent cellular defense against cell death, emphasizing the potential therapeutic significance of simultaneously targeting GD3S in combination with p53 reactivation strategies.

### Limitations of the study

While this study offers important insights into the interplay between GD3S and *TP53* in breast cancer, it underscores the need for further investigation into the mechanisms by which GD3S influences resistance to *TP53*-mediated apoptosis. Our previous findings linked GD3S with the FAK/AKT/mTOR signaling pathway, which promotes cell proliferation by upregulating mitochondrial activity, but the precise mechanism of GD3S-mediated mitochondrial regulation remains unclear [7, 55, 58–60]. We also report that both WT and mutant p53 interact with the GD3S promoter, but the differential transcriptional regulation by p53 mutants is not fully explained. Future studies should explore whether additional factors, such as other transcription factors like NF-κB or co-regulators, contribute to regulating GD3S expression across different p53 variants.

## Supplementary information

Supplementary Figures

Supplementary Table 1

Supplementary Table 2

Supplementary Table 3

Supplementary Table 4

Supplementary Table 5

Supplementary Methodology

## Data Availability

This paper does not report original code. RNA-sequencing data are deposited in NCBI’s Gene Expression Omnibus (GEO) and are accessible through the GEO series accession code GSE248670. Additional raw data supporting the findings of this study are available on Figshare under the DOI (10.6084/m9.figshare.27600672). Data will be made available upon reasonable request to the corresponding author [66].
