# Supplementary material for: GD3 synthase drives resistance to p53-induced apoptosis in breast cancer by modulating mitochondrial function
Source: Oncogene. 2025 May 17;44(30):2646–61. doi: 10.1038/s41388-025-03432-x (PMC12277176; doi:10.1038/s41388-025-03432-x)
Supplement: Supplementary file 1 — Supplementary Figures [file 41388_2025_3432_MOESM1_ESM.pdf]

Supplementary figure 1:

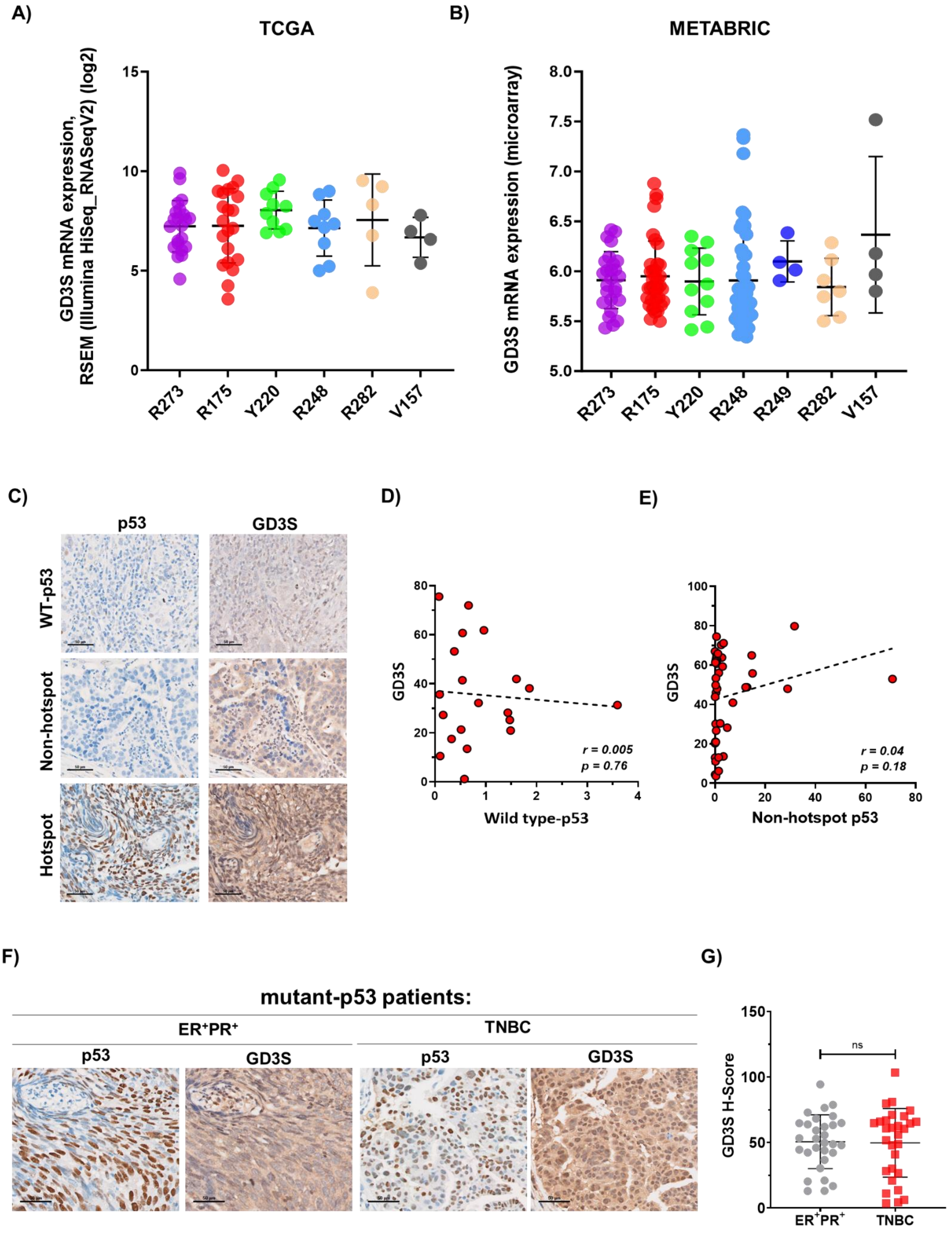

Supplementary Figure S1: (A, B) In the TCGA and METABRIC datasets, GD3S mRNA expression did not differ significantly between breast cancer patients with different p53 hotspot mutations. RSEM, RNA-seq by expectation-maximization. **(C)** Representative IHC images (a second set of representative images from patient's tissue samples) of p53 and GD3S expression in archived FFPE samples from breast cancer patients with WT p53. **(D, E)** Pearson correlation coefficient analysis revealed linear correlations between GD3S and p53 protein expression in patients with WT p53 (r = 0.005) and patients with non-hotspot p53 mutations (r = 0.04). **(F, G)** Among patients with p53 mutations, p53 and GD3S expression did not differ significantly between patients with ER^+PR^+ breast cancer and those with TNBC. ns, not significant.

Supplementary figure 2:

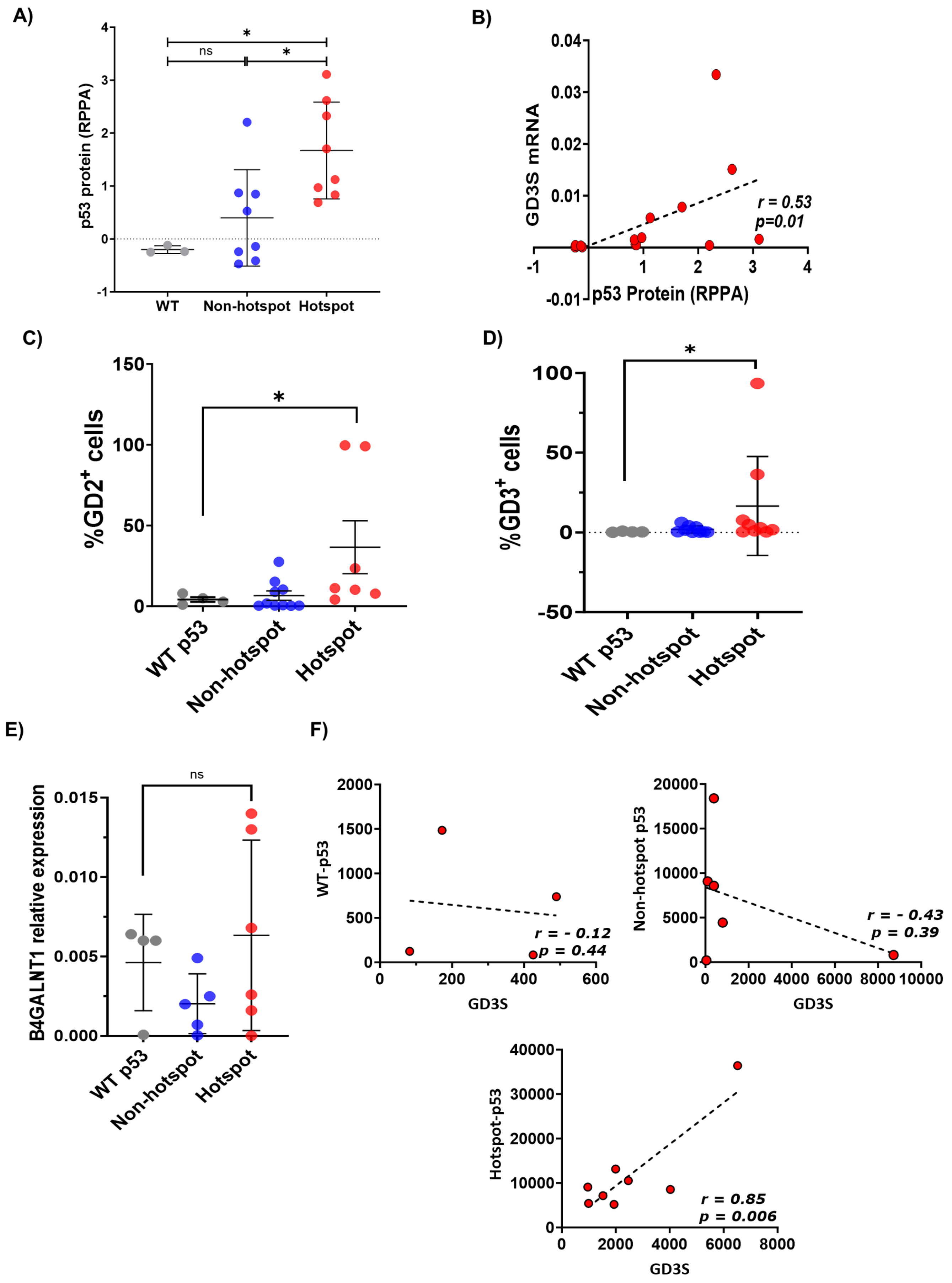

Supplementary Figure S2: (A) RPPA-measured p53 expression levels for each cell line were acquired from the MD Anderson Cell Lines Project and stratified according to p53 mutation status. **(B)** Pearson correlation coefficient analysis revealed a linear correlation between GD3S mRNA and protein levels in different breast cancer cell lines (r = 0.53; P = 0.01). **(C, D)** Flow cytometry revealed that cell lines with p53 hotspot mutations had a significantly higher proportion of $GD2^+$ (C) and $GD3^+$ (D) cells than cell lines with WT p53 or non-hotspot p53 mutations did (P = 0.02 and P = 0.01, respectively, for non-hotspot). **(E)** The relative mRNA expression of GD2 synthase (*B4GALNT1*) did not differ significantly between cell lines with hotspot p53 mutations and those with WT p53. **(F)** Pearson correlation coefficient analysis revealed linear correlations between GD3S and p53 protein expression in cell lines with WT p53 (r = –0.12; P = 0.44), cell lines with non-hotspot p53 mutations (r = –0.43; P =0.39), and cell lines with hotspot p53 mutations (r = 0.85; P = 0.006).

Supplementary figure 3:

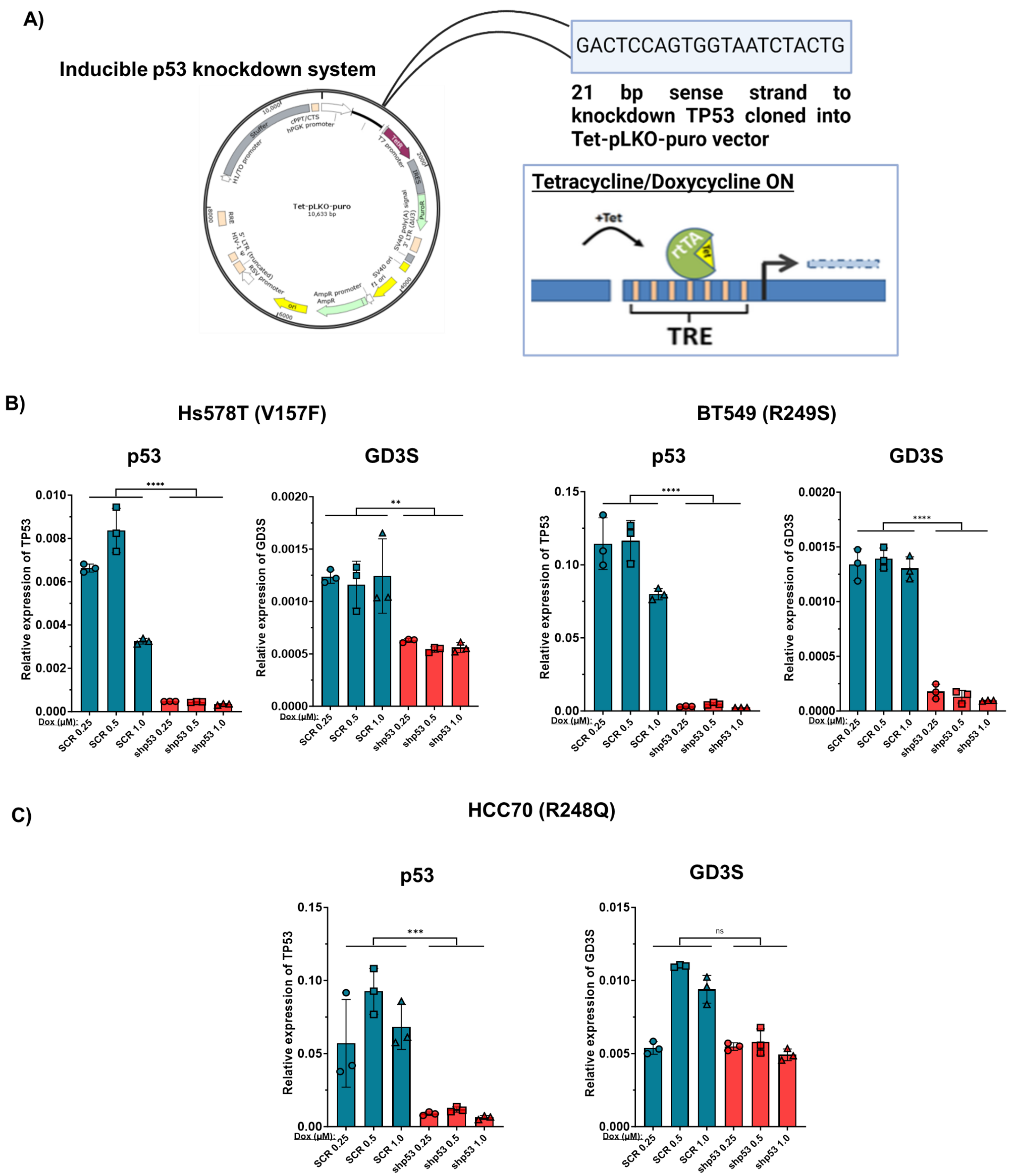

Supplementary Figure S3: (A) Diagram illustrating the inducible p53 knockdown system. A 21-nucleotide p53-siRNA is inserted into the tetracycline-pLKO-puro vector and stably transfected into different breast cancer cells. **(B)** Treatment with increasing concentrations of doxycycline (0.25, 0.5, and 1 µM) resulted in the significant knockdown of p53 and reduction of GD3S expression in Hs578T (V157F) and BT549 (R249S) stable BC cell lines. ***P < 0.01; ****P < 0.0001*. SCR refers to the scramble shRNA for p53 knockdown plasmid. **(C)** HCC70 cells with inducible p53 knockdown were treated with different doses of doxycycline (Dox; 0.25, 0.5, and 1 µM) to induce p53 knockdown, which did not result in a significant decrease in GD3S expression. SCR refers to the scramble shRNA for p53 knockdown plasmid.

Supplementary figure 4:

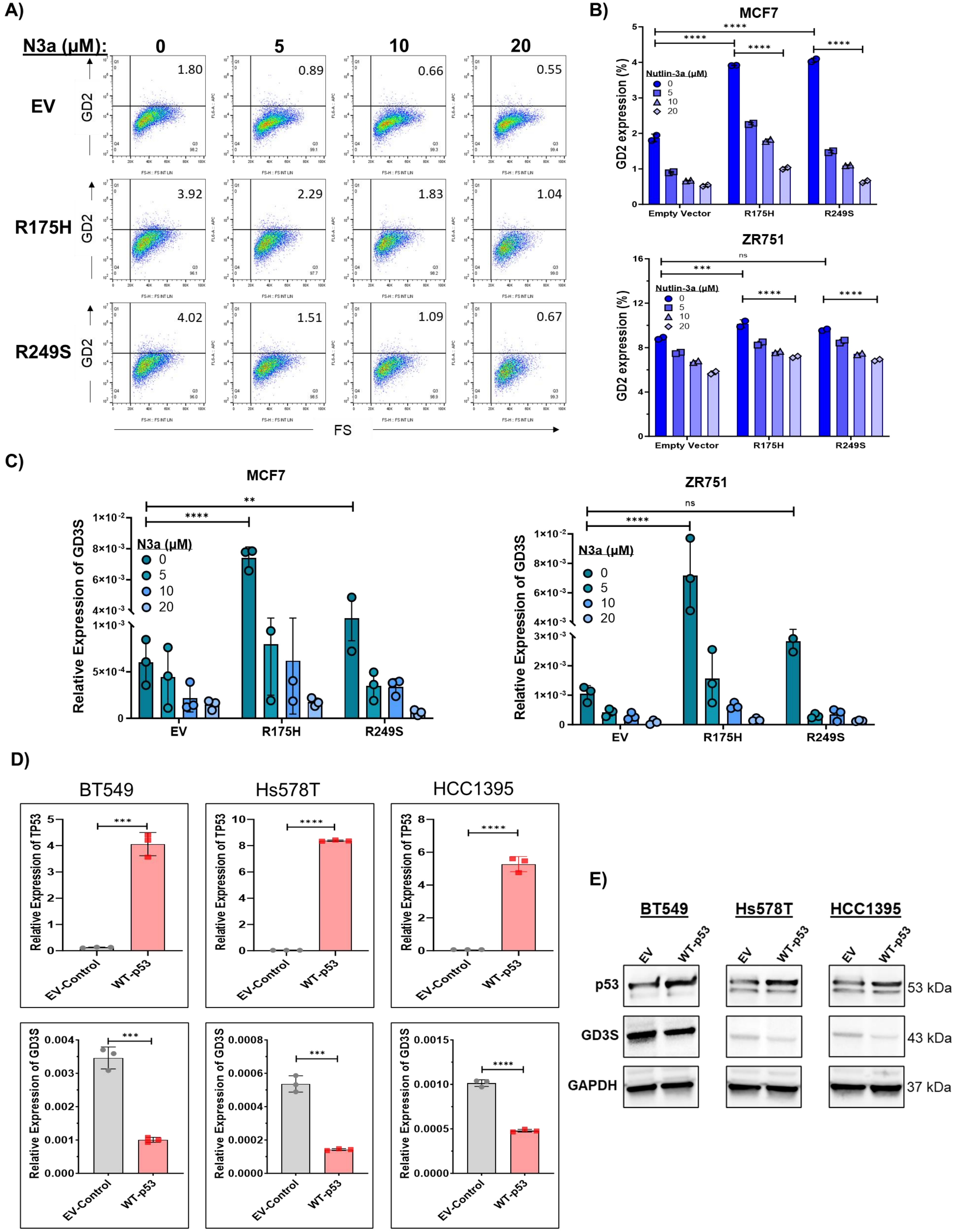

Supplementary Figure S4: (A–C) Flow cytometry and quantitative-PCR was performed to investigate GD2 and GD3S expression in MCF7 and ZR751 cells. These cells were transfected with different plasmids encoding p53 mutants (R175H and R249S) or with EV control plasmids and then treated with different doses of nutlin-3a (N3a; 0, 5, 10, and 20 µM). Cells transfected with the mutant p53 plasmids had substantially higher levels of GD2 and GD3S, and N3a treatment reversed this effect, leading to a dose-dependent reduction in GD2 levels and GD3S mRNA expression. $^{**}P < 0.01$; $^{***}P < 0.001$; $^{****}P < 0.0001$. **(D, E)** In BT549, Hs578T, and HCC1395 cells, inducing the overexpression of WT p53 significantly reduced GD3S mRNA and protein expression. $^{***}P < 0.001$; $^{****}P < 0.0001$.

Supplementary figure 5:

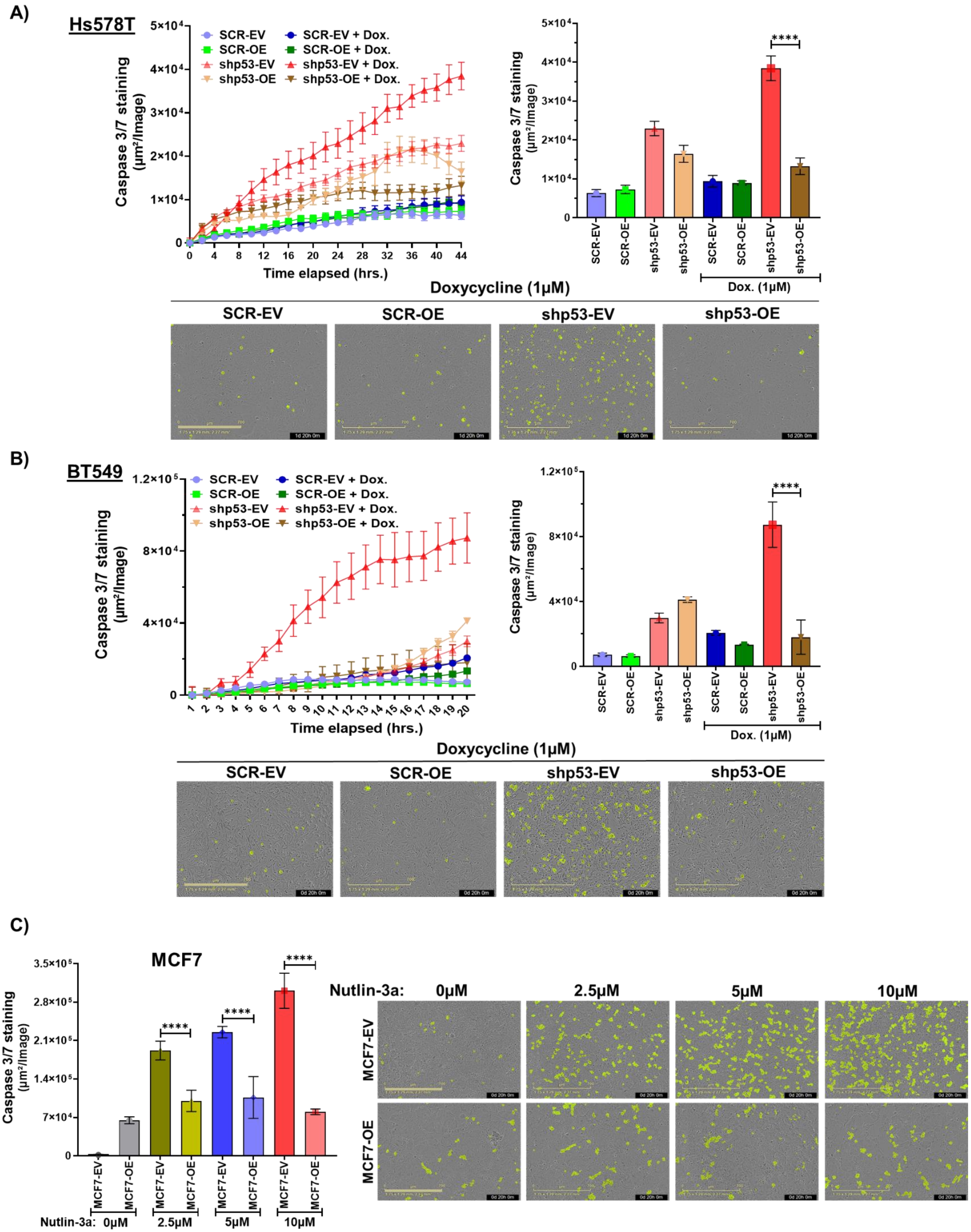

Supplementary Figure S5: (A, B) Hs578T and BT549 cells were modified to have inducible p53 knockdown and GD3S overexpression. To investigate the effect of p53 knockdown and GD3S overexpression, cells were treated with 1 µM doxycycline (Dox) and assessed for Caspase-3/7 staining using IncuCyte live-cell imaging. The graphs show time-dependent Caspase-3/7 green fluorescence staining (μm^2/image) for Hs578T (44 hours) and BT549 (20 hours), while the representative IncuCyte images were taken at the respective time points. p53 knockdown significantly increased Caspase-3/7 activity in both Hs578T and BT549 cells, whereas GD3S overexpression reversed this effect. (**C)** MCF7 cells expressing either an empty vector (MCF7-EV) control or GD3S overexpression (MCF7-OE) were treated with increasing concentrations of Nutlin-3a (2.5, 5, and 10 µM). The effect on Caspase activity was measured using Caspase-3/7 fluorescence in IncuCyte. Nutlin-3a treatment significantly increased Caspase-3/7 activity in MCF7-EV cells, whereas GD3S overexpression reversed this effect. Scale bar: 700 µm. ****$P < 0.0001$.

Supplementary figure 6:

A)

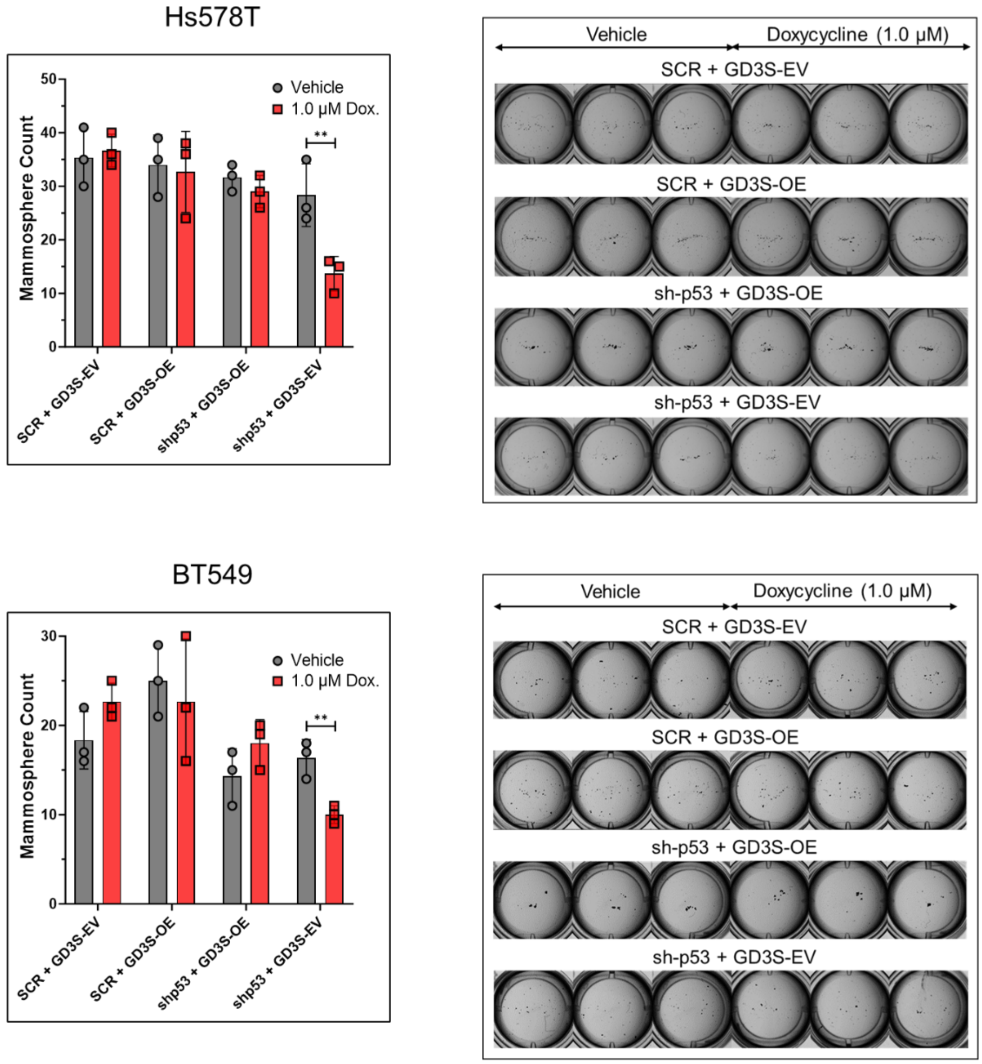

B)

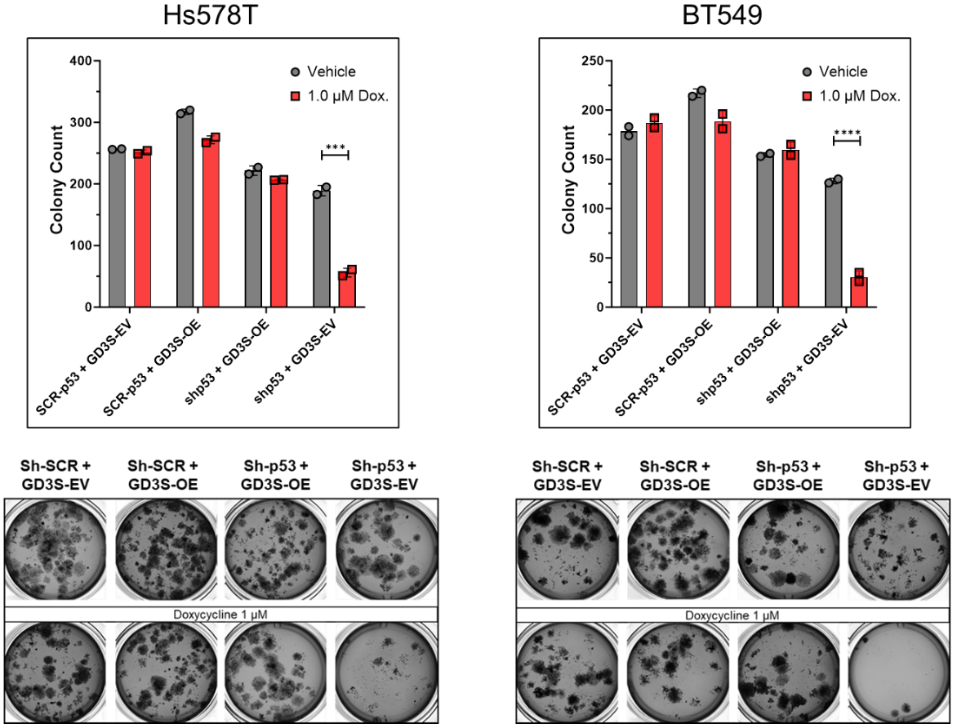

Supplementary Figure S6: (A, B) Hs578T and BT549 cells were modified to have inducible p53 knockdown and GD3S overexpression. To investigate the impact of p53 knockdown and GD3S overexpression, we treated the cells with 1 μM doxycycline (Dox) and assessed their mammosphere-forming ability (A) and colony-forming ability (B). p53 knockdown significantly reduced the mammosphere- and colony-forming abilities of both Hs578T and BT549 cells; however, GD3S overexpression reversed this effect. **$P < 0.01$; ***$P < 0.001$; ****$P < 0.0001$.

Supplementary figure 7:

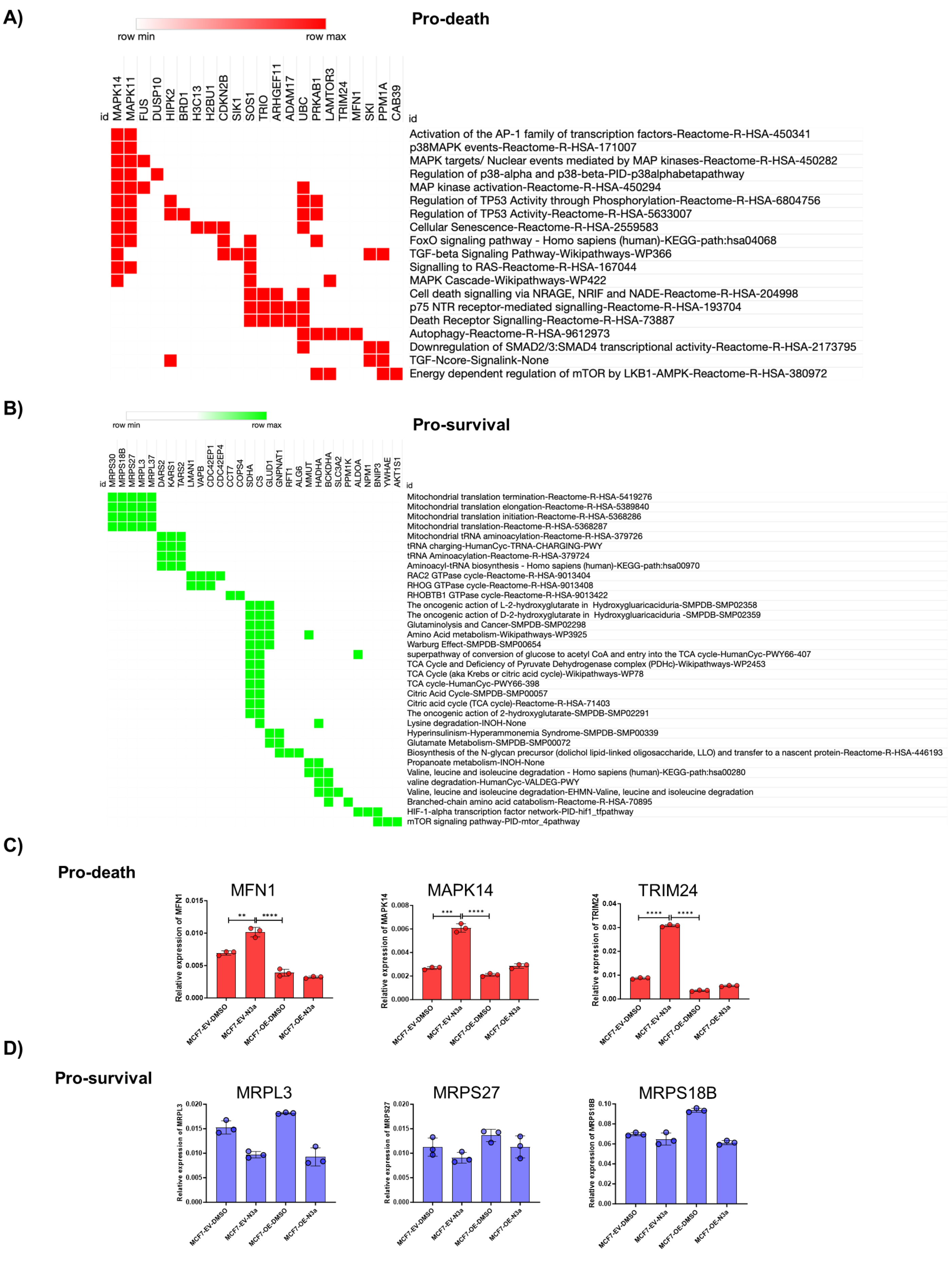

Supplementary Figure S7: (A and B) Pathway-gene matrix for 19 pathways overrepresented by 22 pro-death genes (A), and pathway-gene matrix for 34 pathways overrepresented by 30 pro-survival genes (B). Each row represents one pathway, and each column represents one gene. A green grid indicates the pathway contains the gene as a member. The order of entities was decided by bi-hierarchical clustering using average linkage and Jaccard index as similarity metric. **(C and D)** Verification of RNA-sequencing data through quantitative gene expression (qPCR) analysis revealed elevated expression of pro-death genes in MCF7-EV-control cells following N3a treatment compared to MCF7-GD3S-OE cells (C). Overexpression of GD3S resulted in increased expression of pro-survival genes in MCF7 cells relative to control cells (D). $^{**}P < 0.01$; $^{***}P < 0.001$; $^{****}P < 0.0001$.
