# Supplementary material for: GD3 synthase drives resistance to p53-induced apoptosis in breast cancer by modulating mitochondrial function
Source: Oncogene. 2025 May 17;44(30):2646–61. doi: 10.1038/s41388-025-03432-x (PMC12277176; doi:10.1038/s41388-025-03432-x)
Supplement: Supplementary file 2 — Supplementary Table 1 [file 41388_2025_3432_MOESM2_ESM.docx]

**Supplementary Table S1: ER/PR^+^ breast cancer patient characteristics and treatment details.**

| S. No. | GD3S (H-Score) | ER / PR / Her2 status | Tumor grade | Initial treatment (Tx) | Relapse (Y/N) | Follow up treatment (Tx) | Total time under Tx (months) |
| --- | --- | --- | --- | --- | --- | --- | --- |
| Patients with wild-type p53: | | | | | | | |
| WT01 | 21.29 | ER 90% / PR 70% / Her2 0 | NA | Total Mastectomy | Y (2x) | Neoadj. chemo. with Adriamycin & Cyclophosphamide > Taxol > Rad. Tx > Letrozole > Exemestane & Everolimus > Aromasin & Xgeva > Abraxane > Doxil > INV-(2017-0499) M7824 (MSB0011359C) > Rad. Tx > Eribulin > Gemcitabine. | 228 |
| WT02 | 35.66 | ER 90% / PR ─ / Her2 0 | 2 | Neoadj. Chemo. with Taxotere and Cytoxan > total mastectomy | Y (2x) | TC > Tamoxifen > AC > Rad. Tx > C1 Ibrance & Letrozole > Xgeva, Fulvestrant > Eribulin > Navelbine. | 117 |
| WT03 | 20.92 | ER 70% / PR 10% / Her2 0 | 2 | Taxotere & Cytoxan | Y | Arimidex, Faslodex, & denosumab > Exemestane and everolimus > Letrozole & palbociclib > Xeloda > Doxil > Tamoxifen & denosumab > Taxol. | 129 |
| WT04 | 41.47 | ER 100% / PR 15% / Her2 0 | 1 | NA | NA | FAC > Paclitaxel > Arimidex > Rad. Tx > Verzinio > Eribulin > Abraxane > Metastatic | 45 |
| WT05 | 61.81 | ER 95% / PR 30% / Her2 0 | 2 | NA | NA | A randomized double-blind study of LEE011 in combination with letrozole > Denosumab > Palbociclib & Fulvestrant > Faslodex & Palbociclib > Randomized Phase II Trial for Alisertib Alone or Combined with Fulvestrant > PACLitaxel > Capecitabine > Fulvestrant > PF-06873600 (CDK2 inhibitor) + Fulvestrant > INV-(2021-0265) IACS-6274 (IPN60090) | 90 |
| WT06 | 28.19 | ER 90% / PR 60% / Her2 0 | 2 | Taxol > AC > A phase II study of pembrolizumab in combination with hormonal therapy in patients with HR-positive localized IBC who did not achieve a pCR to Neoadj. Chemo. Exemestane & Pembrolizumab. | Y | Ibrance/Faslodex > Everolimus & tamoxifen . Capecitabine > Eribulin > Rad. tx > Gemcitabine. | 46 |
| WT07 | 27.29 | ER 97% / PR 75% / Her2 0 | 3 | NA | NA | Trial of Carboplatin & paclitaxel W/Wo Veliparib (ABT-888) > Zometa > mastectomy > Palbociclib & AI > Rad. tx. > Phase 1B study to assess the safety, tolerability, and clinical activity of Gedatolisib in combination with Palbociclib and either Letrozole or Fulvestrant in women with metastatic or locally advanced/recurrent breast cancer (MBC) > Capecitabine > Doxorubicin and Cyclophosphamide (AC) | 36 |
| WT08 | 41.95 | ER 0% / PR <1% / Her2 0 | 3 | NA | NA | Taxol/carboplatin > AC | 5 |
| WT09 | 13.48 | ER 2% / PR ─ / Her2 2+ | 3 | NA | NA | AC > TCHP > Rad. tx. > Kadcyla > olaparib > Cisplatin > Xeloda > Phase I Prexasertib + LY3023414 (PI3K inhibitor) > radiation therapy | 28 |
| WT10 | 60.73 | ER ─ / PR <1% / Her2 0 | 3 | NA | NA | Taxol/carboplatin > AC | 42 |
| WT11 | 18.73 | ER 90% / PR 90% / Her2 0 | 3 | Neoadj. Chemo. with FAC > Additional Adj. chemo. with FAC > Adj. Tamoxifen | Y | Rad. tx > Ribociclib & Aromatase Inhibitor | 277 |
| WT12 | 32.2 | ER 95% / PR ─ / Her2 0 | 2 | NA | NA | Palbociclib + Letrozole > Pemigatinib (INCB054828) | 15 |
| WT13 | 17.51 | ER ─ / PR 2% / Her2 2+ | 3 | NA | NA | Atezolizumab (Tecentriq) & Abraxane > Olaparib > Dose Dense Doxorubicin & Cyclophosphamide (AC) | 14 |
| WT14 | 1.14 | ER 100% / PR 0% / Her2 1+ | 2 | NA | NA | Goserelin Acetate Implant (Zoladex) > Palbociclib + Anastrozole > Fulvestrant (Faslodex) > INV-(2019-0668) zotatifin (eFT226) > Goserelin Acetate Implant (Zoladex) > Capecitabine | 19 |
| WT15 | 38.16 | ER 90% / PR 95% / Her2 0 | 2 | Mastectomy > Taxol > ddAC > Rad. Tx | Y | Rad. Tx > GSK 525762 & Fulvestrant > Fulvestrant > Alpelisilib & Fulvestrant > Everolimus + Exemestane > Rad. Tx > Xeloda > Fruquintinib > Eribulin | 85 |
| WT16 | 31.33 | ER 50% / PR ─ / Her2 0 | 3 | NA | NA | Surgery > ddAC > Taxol & Carboplatin > Single agent Taxol > Eribulin > Ixempra | 12 |
| WT17 | 53.22 | ER 95% / PR 95% / Her2 0 | 1 | NA | NA | Surgery > Rad. Tx > Letrozole > Denosumab > Fulvestrant + Letrozole > Palbociclib > Tamoxifen > Palbociclib & Exemestane > Alpelsib | 111 |
| WT18 | 25.23 | ER 10% / PR ─ / Her2 0 | 2 | Lumpectomy > Docetaxel > Cyclophosphamide > Rad. Tx > Arimidex | Y | Paclitaxel > Mastectomy > Atezolizumab & Eribulin > Eribulin > Xeloda > Sacituzumab govitecan-hziy | 87 |
| WT19 | 71.99 | ER 100% / PR 0% / Her2 0 | NA | NA | NA | Paclitaxel > Letrozole + Palbociclib + Xgeva > Eribulin > Capecitabine + Zometa > Liposomal doxorubicin | 56 |
| WT20 | 75.58 | ER 91% / PR 8.6% / Her2 0 | 2 | NA | NA | Paclitaxel > Denosumab > Letrozole + Palbociclib > ddCapecitabine | 37 |
|  |  |  |  |  |  |  |  |
| Patients with mutant p53: | | | | | | | |
| MT01 | 53.37 | ER 80% / PR 75% / Her2 0 | 1 | NA | NA | Metastatic > Palbociclib & Letrozole > Palbociclib + Exemestane | 27 |
| MT02 | 42.25 | ER ─ / PR 10% / Her2 0 | 3 | NA | NA | ddDoxorubicin and Cyclophosphamide (AC) > Paclitaxel (TAXOL) > Rad. tx > Abraxane > Metastatic | 18 |
| MT03 | 94.39 | ER 90% / PR 5% / Her2 0 | 2 | NA | NA | Neoadj. Letrozole > OSF AC > Paclitaxel > Rad. Tx > Anastrozole | 11 |
| MT04 | 46.02 | ER 0% / PR 2% / Her2 0 | NA | NA | NA | Chemo. > Taxol | 14 |
| MT05 | 48.78 | ER 100% / PR 40% / Her2 2+ | 3 | NA | NA | Denosumab > Letrozole & Ibrance > Taxol > Doxil | 21 |
| MT06 | 30.38 | ER 20% / PR 80% / Her2 0 | 3 | NA | NA | ddAC > Taxol with Carboplatin > Rad. Tx > Xeloda > Liposomal Doxorubicin and Everolimus | 19 |
| MT07 | 63.85 | ER 95% / PR 5% / Her2 0 | 2 | NA | NA | Anastrozole > Taxol > Xgeva > Faslodex > Continued Xgeva > Ibrance > Continued Faslodex & Xgeva > Rad. Tx > Tamoxifen > Rad. Tx > Exemestane > Rad. Tx | 31 |
| MT08 | 43.89 | ER 50% / PR 1% / Her2 0 | 3 | NA | NA | Zoladex > Neoadj. Chemo. dose dense AC > ddTaxol > Anastrazole > Ixempra > Faslodex/palbociclib > Zoladex > Rad. Tx | 22 |
| MT09 | 47.96 | ER 80% / PR ─ / Her2 0 | 3 | NA | NA | Neoadj. Chemo. > dose dense AC/Taxol > Rad. Tx > Anastrozole > Carboplatin + Gemcitabine > Atezolizumab (Tecentriq) & Abraxane 5/19, | 18 |
| MT10 | 63.33 | ER 90% / PR 70% / Her2 2+ | 2 | NA | NA | Herceptin + pertuzumab + taxol > Rad. Tx > Pertuzumab and Trastuzumab (Herceptin) > Kadcyla > Neratinib/Capecitabine > Enhertu > Margetuximab | 32 |
| MT11 | 36.75 | ER 98% / PR ─ / Her2 0 | 3 | NA | Y | Tax. > Palbociclib & Letrozole > Alpelisib & Fulvestrant > Gemcitabine | 60 |
| MT12 | 13 | ER <1% / PR <1% / Her2 0 | 3 | NA | NA | Carboplatin + Paclitaxel > AC > Carbo + taxol > Eribulin > Rad. Tx | 13 |
| MT13 | 49.82 | ER 99% / PR <1% / Her2 3+ | 2 | NA | NA | Taxol + HP > HP + Arimidex > Taxol > Abraxane > Metastatic > Tamoxifen > Capecitabine > Trastuzumab (Herceptin) > Rad. Tx > Eribulin > Palbociclib and Fulvestrant | 31 |
| MT14 | 52.95 | ER 3% / PR 0% / Her2 1+ | 3 | ddDoxorubicin and Cyclophosphamide (AC) > Panitumumab & Paclitaxel weekly + Carboplatin every 3 weeks > Capecitabine > Rad Tx | Y | Metastatic > Sacituzumab govitecan-hziy (Trodelvy) | 22 |
| MT15 | 64.78 | ER 12% / PR ─ / Her2 1+ | 3 | NA | NA | Neoadj. Chemo. > ddAC > Paclitaxel > Progression breast and lymph nodes > Carboplatin + taxol > Copanlisib & nivolumab > Sacituzumab (Trodelvy) > Capecitabine and ixabepilone (Ixempra) > Eribulin | 18 |
| MT16 | 76.51 | ER 50% / PR 5% / Her2 0 | 3 | NA | NA | Taxol > ddAC > Xeloda > Denosumab > Eribulin > Fulvestrant & Abemaciclib | 27 |
| MT17 | 42.36 | ER 89% / PR 3.2% / Her2 1+ | 3 | NA | NA | Taxol neoadjuvant > AC > Tamoxifen > Zometa > Faslodex & Ibrance > Alisertib > Capecitabine > Everolimus & Exemestane > Paclitaxel | 39 |
| MT18 | 59.26 | ER 85% / PR 25% / Her2 0 | 3 | NA | NA | Taxol > AC > Rad. Tx > Arimidex > Palbociclib & Fulvestrant > Everolimus & Exemestane > INV-(2020-0621) PF-07104091 > Capecitabine > Eribulin + Gemcitabine > Rad. Tx | 58 |
| MT19 | 72.98 | ER 95% / PR 5% / Her2 0 | 3 | NA | NA | ddAC chemotherapy > ddTaxol > Zoladex + Aromasin > Rad Tx > Ibrance + Fulvestrant + Goserelin > Capecitabine > Eribulin Gemcitabine > Doxorubicin liposomal | 40 |
| MT20 | 62.05 | ER 99% / PR 90% / Her2 0 | 2 | NA | NA | ddAC > Taxol & Carboplatin > Rad. Tx > Capecitabine > Xeloda > Ixabepilone | 19 |
| MT21 | 68.24 | ER 5% / PR 0% / Her2 0 | 3 | NA | NA | AC > Carbo + Taxol + Panitumumab with progression of disease > new metastatic disease > Rad. Tx > Doxorubicin liposomal > NEU: IT Chemo. - Topotecan > Eribulin | 35 |
| MT22 | 44.55 | ER 20% / PR 84% / Her2 0 | 2 | Neoadj. chemo. with Docetaxel, Adriamycin & Cytoxan > Ovarian suppression and Faslodex > Arimidex | Y | Carboplatin & Paclitaxel > Xeloda > Gemzar > Rad. Tx > Letrozole & Abemaciclib | 35 |
| MT23 | 55.78 | ER 1.7% / PR <1% / Her2 2+ | 3 | NA | NA | Cycles of Perjeta/Taxotere/Carboplatin/Herceptin > Perjeta/Herceptin > INV-(2018-0636) DS-1062a | 17 |
| MT24 | 64.95 | ER 7% / PR ─ / Her2 0 | N/A | NA | NA | ddAdriamycin + Cytoxan > Taxol + Carboplatin > Sacituzumab Govotecan | 11 |
| MT25 | 70.23 | ER 17% / PR 12% / Her2 1+ | 2 | NA | NA | Cytoxan > Doxorubicin > Rad. Tx > Paclitaxel > Capecitabine > Sacituzumab govitecan-hziy | 10 |
| MT26 | 78.83 | ER 90% / PR 0% / Her2 1+ | 3 | NA | NA | AC > Taxol > Ixabepilone & Capecitabine Chemo. > Goserelin & Tamoxifen | 26 |
| MT27 | 70.12 | ER 76% / PR 59% / Her2 0 | 3 | NA | NA | AC > Paclitaxel > Rad. Tx > Phase II Study of Pembrolizumab + Hormonal therapy in HR-positive localized IBC Patients with Non-pCR to Neoadj. Chemo. > Tamoxifen > Letrozole > Ovarian suppression > Eribulin & Trastuzumab with Pertuzumab > Doxorubicin & Cyclophosphamide (AC) > Rebastinib (DCC-2036) with PACLitaxel > Alpelisib & Fulvestrant | 24 |
| MT28 | 20.3 | ER 100% / PR 90% / Her2 1+ | 2 | TC > Tamoxifen > Rad. Tx | Y | Palbociclib > Letrozole + Zoladex > Clinical trial Alisertib + Fulvestrant > Capecitabine > Doxil > Everolimus & Exemestane > Taxol > Carboplatin & Gemcitabine | 110 |
| MT29 | 21.04 | ER 99% / PR <1% / Her2 3+ | 2 | NA | NA | Taxol > HP > HP + Arimidex > Taxol (HP on hold) > Abraxane > Metastatic > Tamoxifen > Capecitabine > Trastuzumab > Rad. Tx > Eribulin > Palbociclib & Fulvestrant | 31 |
| MT30 | 13.11 | ER 70% / PR 80% / Her2 0 | 2 | NA | NA | Herceptin + Pertuzumab + Taxol > Rad. Tx > Pertuzumab & Trastuzumab > Kadcyla > Neratinib/Capecitabine > Enhertu > Margetuximab | 32 |

WT = wild-type p53; MT = mutant p53; Neoadj. Chemo. = Neoadjuvant Chemotherapy; Rad. Tx = Radiation treatment; TC = Docetaxel + Cyclophosphamide; AC = Adriamycin & Cyclophosphamide; dd = dose-dense; HP = Herceptin + Perjeta; NA = Not Available.
