# Supplementary material for: GD3 synthase drives resistance to p53-induced apoptosis in breast cancer by modulating mitochondrial function
Source: Oncogene. 2025 May 17;44(30):2646–61. doi: 10.1038/s41388-025-03432-x (PMC12277176; doi:10.1038/s41388-025-03432-x)
Supplement: Supplementary file 3 — Supplementary Table 2 [file 41388_2025_3432_MOESM3_ESM.docx]

**Supplementary Table S2: Characteristics of breast cancer cell lines.**

LAR: Luminal androgen receptor; MSL: Mesenchymal stem like; Immun.: Immunomodulatory; BL1: Basal like 1; BL2: Basal like 2

| ***Cell line*** | ***Molecular***  ***Subtype*** | ***ER/PR/Her2 status*** | ***Hotspot*** | ***p53 mutation*** | ***p53 mutation type*** | ***Domain*** | ***p53 protein expression by RPPA*** | ***% GD3***  ***(mean ± SD)*** | ***% GD2***  ***(mean ± SD)*** |
| --- | --- | --- | --- | --- | --- | --- | --- | --- | --- |
| **MDA-MB-453** | LAR | ─/─/+ | N | Frameshift deletion | Frameshift deletion C terminal | DNA binding | 0.84718 | 0.06 ± 0.01 | 0.28 ± 0.05 |
| **MDA-MB-436** | MSL | ─/─/─ | N | Frameshift insertion | 7bp insertion | DNA binding | -0.41091 | 6.26 ± 0.86 | 27.5 ± 1.35 |
| **HCC1187** | Immun. | ─/─/─ | N | In frame deletion | In frame deletion | DNA binding | 2.2053 | 0.36 ± 0.21 | 0.32 ± 0.07 |
| **SUM159** | MSL | ─/─/─ | N | In frame insertion | In frame insertion | DNA binding | NA | 0.18 ± 0.07 | 15.20 ± 0.60 |
| **HCC1806** | BL2 | ─/─/─ | N | In frame insertion | 2bp insertion | DNA binding | -0.24127 | 0.15 ± 0.03 | 0.69 ± 0.05 |
| **BT20** | BL1 | ─/─/─ | N | K132Q | Missense mutation | DNA binding | NA | 0.38 ± 0.11 | 0.44 ± 0.15 |
| **MDA-MB-231** | MSL | ─/─/─ | N | R280K | Missense mutation | DNA binding | 0.87115 | 4.18 ± 0.11 | 10.40 ± 0.20 |
| **MDA-MB-468** | BL1 | ─/─/─ | Y | R273H | Missense mutation | DNA binding | 2.6164 | 93.45 ± 0.05 | 4.14 ± 0.15 |
| **Hs578T** | MSL | ─/─/─ | Y | V157F | Missense mutation | DNA binding | 0.97037 | 4.87 ± 0.08 | 99.10 ± 0.20 |
| **HCC1143** | BL1 | ─/─/─ | Y | R248Q | Missense mutation | DNA binding | 3.11 | 0.96 ± 0.17 | 11.2 ± 1.06 |
| **HCC70** | BL2 | ─/─/─ | Y | R248Q | Missense mutation | DNA binding | 2.3265 | 36.35 ± 0.95 | 9.85 ± 0.75 |
| **HCC38** | BL2 | ─/─/─ | Y | R273L | Missense mutation | DNA binding | 1.1231 | 1.69 ± 0.13 | 23.5 ± 1.10 |
| **BT549** | MSL | ─/─/─ | Y | R249S | Missense mutation | DNA binding | 0.83432 | 2.82 ± 0.19 | 7.87 ± 0.32 |
| **HCC1395** | BL2 | ─/─/─ | Y | R175H | Missense mutation | DNA binding | 1.7032 | 7.7 ± 0.98 | 99.70 ± 0 |
| **SKBR3** | LAR | ─/─/+ | Y | R175H | Missense mutation | DNA binding | 0.68833 | 0.38 ± 0.02 | 1.38 ± 0.17 |
| **T47D** | LAR | +/+/─ | N | L194F | Missense mutation | DNA binding | 0.52726 | 1.47 ± 0.34 | 8.90 ± 0.42 |
| **SUM149** | BL2 | ─/─/─ | Y | M273I | Missense mutation | DNA binding | NA | 0.36 ± 0.16 | 0.22 ± 0.10 |
| **BT474** | LAR | +/+/+ | Y | E285K | Missense mutation | DNA binding | 1.2302 | 0.31 ± 0.07 | 0.23 ± 0.08 |
| **HCC1937** | BL1 | ─/─/─ | N | Nonsense mutation | Nonsense mutation | NLS | -0.14072 | 3.44 ± 0.21 | 1.72 ± 0.34 |
| **HCC1599** | BL1 | ─/─/─ | N | Splice site mutation | Splice site mutation | Intron mutation | -0.47118 | NA | 0.42 ± 0.07 |
| **DU4475** | Immun. | ─/─/─ | NA | None | Wild type | NA | -0.25593 | 0.29 ± 0 | 0.97 ± 0.07 |
| **MCF-7** | LAR | +/+/─ | NA | None | Wild type | NA | -0.24868 | 0.34 ± 0.09 | 3.99 ± 0.37 |
| **ZR751** | LAR | +/+/─ | NA | None | Wild type | NA | -0.11708 | 0.049 ± 0.01 | 4.87 ± 0.51 |
| **HIM3** | Unknown | ─/─/─ | NA | None | Wild type | NA | NA | 0.82 ± 0.05 | 7.96 ± 0.52 |

LAR: luminal androgen receptor; MSL: mesenchymal stem-like; Immun.: immunomodulatory; BL1: basal-like 1; BL2: basal-like 2.
