# Supplementary material for: GD3 synthase drives resistance to p53-induced apoptosis in breast cancer by modulating mitochondrial function
Source: Oncogene. 2025 May 17;44(30):2646–61. doi: 10.1038/s41388-025-03432-x (PMC12277176; doi:10.1038/s41388-025-03432-x)
Supplement: Supplementary file 4 — Supplementary Table 3 [file 41388_2025_3432_MOESM4_ESM.docx]

**Supplementary Table S3: Effects of p53 knockdown on cell survival and GD3S expression in breast cancer cell lines harboring hotspot p53 mutations.**

LAR: Luminal androgen receptor; MSL: Mesenchymal stem like; Immun.: Immunomodulatory; BL1: Basal like 1; BL2: Basal like 2

| Cell line | Hotspot p53 mutation | Effect of p53 knockdown on cell survival | Effect of p53 knockdown on GD3S expression |
| --- | --- | --- | --- |
| **Hs578T** | V157F | Complete cell death | Significant decrease |
| **HCC38** | R273L | Complete cell death | Significant decrease |
| **BT549** | R249S | Complete cell death | Significant decrease |
| **HCC1395** | R175H | Complete cell death | Significant decrease |
| **MDA-MB-468** | R273H | No cell death | No effect |
| **HCC70** | R248Q | Minimal cell death | No effect |
| **HCC1143** | R248Q | Minimal cell death | No effect |
