# Supplementary material for: GD3 synthase drives resistance to p53-induced apoptosis in breast cancer by modulating mitochondrial function
Source: Oncogene. 2025 May 17;44(30):2646–61. doi: 10.1038/s41388-025-03432-x (PMC12277176; doi:10.1038/s41388-025-03432-x)
Supplement: Supplementary file 5 — Supplementary Table 4 [file 41388_2025_3432_MOESM5_ESM.docx]

**Supplementary Table S4: TaqMan primers used in qPCR.**

LAR: Luminal androgen receptor; MSL: Mesenchymal stem like; Immun.: Immunomodulatory; BL1: Basal like 1; BL2: Basal like 2

| Serial No. | Gene | Primer ID |
| --- | --- | --- |
| 1. | GD3S (ST8SIA1) | Hs00268157_m1 |
| 2. | GD2S (B4GALNT1) | Hs01110791_g1 |
| 3. | P53 | Hs01034249_m1 |
| 4. | MFN1 | Hs00966851_m1 |
| 5. | MAPK14 | Hs01051152_m1 |
| 6. | TRIM24 | Hs00234363_m1 |
| 7. | MRPL3 | Hs00246665_m1 |
| 8. | MRPS27 | Hs00372159_m1 |
| 9. | MRPS18B | Hs00204096_m1 |
| 10. | GAPDH | Hs002758991_g1 |
