# Supplementary material for: GD3 synthase drives resistance to p53-induced apoptosis in breast cancer by modulating mitochondrial function
Source: Oncogene. 2025 May 17;44(30):2646–61. doi: 10.1038/s41388-025-03432-x (PMC12277176; doi:10.1038/s41388-025-03432-x)
Supplement: Supplementary file 6 — Supplementary Table 5 [file 41388_2025_3432_MOESM6_ESM.docx]

**Supplementary Table S5: Primers for the GD3S upstream promoter region used in ChIP-qPCR.**

LAR: Luminal androgen receptor; MSL: Mesenchymal stem like; Immun.: Immunomodulatory; BL1: Basal like 1; BL2: Basal like 2

| Primer | Sequence | Amplicon size, bp |
| --- | --- | --- |
| Primer 1 |  |  |
| FWD | TATCCAAAGCCCAGGCAA | 207 |
| REV | CCCAGAAATCTCTGTCCTACTT |  |
|  |  |  |
| Primer 2 |  |  |
| FWD | CTGATTTACCTAAGTAGGACAGAGATT | 204 |
| REV | CTGGTACCTTTGTCTAGCATCC |  |
|  |  |  |
| Primer 3 |  |  |
| FWD | TCAGTGGAGACGGATGCTA | 195 |
| REV | CATAGCTCGGGTACCTCCTA |  |
|  |  |  |
| Primer 4 |  |  |
| FWD | AATGGCTAACAGCGACTCTC | 188 |
| REV | AACACTAGACCTCAAGATGTCAC |  |
|  |  |  |
| Primer 5 |  |  |
| FWD | TCCAAGGCAATGTGACATCTT | 192 |
| REV | ACACACACACACACACACA |  |
|  |  |  |
| Primer 6 |  |  |
| FWD | TGTGTGTGTGTGTGTGTGT | 220 |
| REV | GCTAATATCCTGATCCTTCGACAA |  |
|  |  |  |
| Primer 7 |  |  |
| FWD | GCCACACTCAGGGACTGATA | 142 |
| REV | GCCTCCTTCGACCCGAT |  |
|  |  |  |
| Primer 8 |  |  |
| FWD | GGGTCGAAGGAGGCAATTT | 143 |
| REV | AAGAACTCGCTCTCATCAGTTAAT |  |
|  |  |  |
| Primer 9 |  |  |
| FWD | TTTCCAAGGACTTGGTGCGG | 154 |
| REV | CTCCTAAACATGTGGCCCGT |  |
|  |  |  |
| Primer 10 |  |  |
| FWD | ACGGGCCACATGTTTAGG | 176 |
| REV | GGGTGAAGTCACGATCTATGG |  |
|  |  |  |
| Primer 11 |  |  |
| FWD | CCCTAGAAAGAAATCCTTGGAAA | 173 |
| REV | CACAAAGCTAGGCGAAGTG |  |
|  |  |  |
|  |  |  |
|  |  |  |
|  |  |  |
| GAPDH |  |  |
| FWD | CCCTTCATACCCTCACGTATTC |  |
| REV | ATGACAAGCTTCCCGTTCTC | 105 |
|  |  |  |
