# Supplementary material for: GD3 synthase drives resistance to p53-induced apoptosis in breast cancer by modulating mitochondrial function
Source: Oncogene. 2025 May 17;44(30):2646–61. doi: 10.1038/s41388-025-03432-x (PMC12277176; doi:10.1038/s41388-025-03432-x)
Supplement: Supplementary file 7 — Supplementary Methodology [file 41388_2025_3432_MOESM7_ESM.docx]

**SUPPLEMENTARY METHODS**

**Animals and tumor xenografts**

All animal experiments were approved by the Institutional Animal Care and Use Committee (IACUC) of The University of Texas MD Anderson Cancer Center (UTMDACC) and conducted in compliance with state and federal regulations. Six- to eight-week-old female NSG mice (NOD.Cg-Prkdc Il2rg/SzJ) were obtained from Jackson Laboratory (Bar Harbor, ME) and housed in a pathogen-free vivarium at the North Campus Animal Care facility, Department of Veterinary Medicine & Surgery (DVMS), UTMDACC, Houston. For xenograft studies (10 mice per group), we ensured >90% power to detect differences such as 20% vs. 40% survival at a significance level of 0.05. The Benjamini–Hochberg procedure (α = 0.01) was applied to control for multiple testing, maintaining ≥82% power, even with potential reductions in sample size or effect size variations. Mice were randomly assigned to treatment groups, and the researcher measuring tumor size was blinded to group allocations. To promote estrogen receptor (ER^+^) progesterone receptor (PR^+^) tumors, mice received estradiol in drinking water (8 µg/mL) before orthotopic transplantation of 3 × 10⁶ MCF7-EV-control or MCF7-GD3S-OE cells into the mammary fat pad. Once tumors reached 100 mm³, mice were randomized into size-matched treatment groups and received bi-weekly intraperitoneal injections of 5 mg/kg Nutlin-3a or vehicle control for 7 weeks. Tumor response and survival were monitored throughout the study. Nutlin-3a stock solution was prepared in DMSO, and injection solutions were diluted in 30% DMSO in sterile PBS.

**Human samples for Immunohistochemistry**

The analysis of patient samples was conducted in accordance with all relevant regulations. Formalin-fixed paraffin-embedded (FFPE) archived primary breast tumor tissues, expressing either wild-type (WT) or mutant p53, were obtained from the Tissue Bank at the University of Texas MD Anderson Cancer Center, under approval from MD Anderson’s Institutional Review Board (Protocol #PA19-0732). The *TP53* expression status in all breast cancer samples was determined using next-generation sequencing by the Sheikh Khalifa Bin Zayed Al Nahyan Institute for Personalized Cancer Therapy (IPCT) at MD Anderson Cancer Center.

**Cell lines**

A panel of breast cancer cell lines were purchased from ATCC (Manassas, VA) and cultured at 37°C in a 5% CO2 incubator less than 6 months after revival, per ATCC recommendations, in either Roswell Park Memorial Institute medium or Dulbecco’s modified Eagle’s medium containing 10% fetal bovine serum (Gibco) and 1% penicillin/streptomycin (Mediatech, Corning Inc., Manassas, VA). SUM159, SUM149 (TNBC) and the non-TNBC cell lines MCF7 and ZR751 were a kind gift from Dr. Naoto Ueno’s laboratory at MD Anderson Cancer Center. The patient-derived xenograft–derived TNBC cell line HIM3 was a kind gift from Dr. Piwnica-Worms’s laboratory at MD Anderson.

**Flow cytometry**

Breast cancer cells were subjected to trypsin and washed once with phosphate-buffered saline (PBS). The cells were then incubated for 30 min with 4 μL/reaction of allophycocyanin-conjugated purified anti-human ganglioside GD2 antibody (357302, BioLegend). For GD3 expression analysis, cells were stained with 3 μL/reaction of unconjugated anti-GD3 antibody (clone R24; MABC1112, Millipore Sigma) for 30 min on ice and then washed twice with PBS. For secondary staining, the cells were incubated for 30 min with 100 μL of diluted goat anti-mouse antibody conjugated to Alexa Fluor 647 (A21235, Thermo Fisher Scientific). Staining with 4’, 6-diamino-2-phenylindole (DAPI; D1306, Thermo Fisher Scientific) was used to exclude dead cells. Allophycocyanin-conjugated purified mouse IgG2A antibody (400219, BioLegend) was used as an isotype control to account for nonspecific antibody binding. After incubation, the cells were washed once with PBS containing 0.5 μg/mL DAPI and analyzed with an LSR II (BD Biosciences) or Gallios (Beckman Coulter) flow cytometer. Ten thousand events were acquired for each sample. All experiments were performed in duplicate, and all flow cytometry data were analyzed with FlowJo software (Version 10, Ashland, OR).

**MDM2 inhibition in cell lines**

Breast cancer cells expressing WT p53 were plated in 6-well plates (Corning Life Sciences) at 5 × 10^5^ cells/well and incubated overnight. The small-molecule MDM2 inhibitor nutlin-3a was purchased from Millipore sigma (SML0580) and stored at –20°C. Serial dilutions of nutlin-3a were prepared based on the drug’s IC50 value (5 µM) and stored in 15-mL Falcon tubes. For each breast cancer cell line, we compared five concentrations of nutlin-3a (1.25 µM, 2.5 µM, 5 µM, 10 µM, and 20 µM) to no treatment to determine its effect on MDM2 inhibition. The culture medium was then aspirated, and the cells were re-suspended in a medium containing the respective drug concentration for 72h.

**Inducible p53 knockdown and GD3S overexpression**

shRNA lentiviral vectors (NM_000660, XM_011527242; clone ID: TRCN0000003318; vector: pLKO.1) and lentiviral pLKO.1 empty vector (EV; cat.# RHS4080) were purchased from GE Dharmacon. HEK293T cells were transfected with each p53 shRNA construct along with the packaging vectors pMD2.G and psPAX2 (Addgene) using jetPRIME transfection reagent (Polyplus) according to manufacturer’s guidelines. Seventy-two hours after transfection, the lentivirus-containing medium was collected and then incubated with target cells for 24 h. The transduced cells were selected using puromycin (0.5 µg/mL) for 3 days, and p53 knockdown efficacy was determined by Western blotting.

To develop a doxycycline-inducible p53 knockdown system, vectors including TET-pLKO-puro (Plasmid# 21915) and TET-pLKO-puro-scrambled (Plasmid# 47541) plasmids were purchased from Addgene. A 21-nucleotide sense strand that targets p53 (GACTCCAGTGGTAATCTACTG) was cloned into the inducible vector TET-pLKO-puro plasmid at MD Anderson’s Functional Genomics Core. Plasmid DNA was extracted using a Midiprep kit (740410, Takara Bio USA Inc.). Inducible cell lines were generated, and p53 knockdown was induced by treating cells with 1 µM doxycycline hydrochloride (D3447, Millipore Sigma) for 48 h. To determine the effect of GD3S overexpression in BC cells, we used the lentivirus packaging method described above and ST8SIA1-plenti-III-CMV (LV701751; Applied Biological Materials) and EV control plenti-III-Blank (LV587; Applied Biological Materials) plasmids to generate stable clones of MCF7 and ZR751 cells. In addition, to determine the effect of GD3S overexpression in cells with the doxycycline-inducible p53 knockdown system, we used the lentivirus packaging method and ST8SIA1-pLX304 (EX-OL01535-LX304; Genecopoeia) and EV control pLX304 plasmids to generate stable cells, which were selected using blasticidin (10 µg/mL) for 5 days. GD3S expression was assessed with quantitative polymerase chain reaction (qPCR). We used jetPRIME reagent according to the manufacturer’s guidelines to transiently transfect different BC cell lines with plasmids encoding mutant p53 (pLenti6/V5-p53_R249S and pLenti/V5-p53_R175H; Addgene) or luciferase (pLL-ST8SIA1-luciferase-T2A-puro, System Biosciences).

**Western blotting**

Cells (3 × 10^6^) were subjected to lysis. Proteins were harvested from the cell lysates using Laemmli buffer containing 50 μL/mL β-mercaptoethanol. Protease and phosphatase inhibitor cocktail was added to the protein lysates at a 1:100 ratio, and 15 μL of each protein sample was loaded onto 4–15% Mini-PROTEAN TGX precast gels for separation (c4561086, Bio-Rad Laboratories). The proteins were subsequently transferred onto a polyvinylidene fluoride membrane, which was then blocked with 5% milk in TBS-T (0.05% Tween-20 in PBS) to prevent the non-specific binding of antibodies. The membranes were incubated with primary antibodies against p53 and ST8SIA1 in PBS-T with 1% milk at 4°C overnight. The membranes were then incubated with goat anti-rabbit (31460, Thermo Fisher Scientific) or goat anti-mouse (31430, Thermo Fisher Scientific) horseradish peroxidase (HRP)-conjugated secondary antibodies. An enhanced chemiluminescence Western blotting substrate kit (32106, Thermo Fisher Scientific) was used for the detection of HRP-conjugated secondary antibodies. The membranes were washed 3 times with TBS-T and imaged using a ChemiDoc MP (Bio-Rad Laboratories) imaging system. All protein quantification was performed using Image Lab software (Bio-Rad Laboratories).

**Immunohistochemistry**

IHC analyses of GD3S and p53 expression were performed with rabbit anti-human polyclonal ST8SIA1/GD3S (24918-1-AP, Proteintech) and mouse anti-human monoclonal p53 (48818, Cell Signaling Technology, Danvers, MA) antibodies. We also performed IHC analyses of p53 and GD3S expression in xenograft tissues derived from MCF7 cells (the methods used to generate the xenografts are described later in the Methods section). Briefly, 4- to 6-µm-thick tissue sections were prepared using a microtome, placed onto positively charged slides, and dried at 56°C overnight. Subsequently, the slides were subjected to deparaffinization and rehydration by sequential immersion in xylene and graded ethanol solutions. The slides were exposed to 3% H_2_O_2_ for 10 min to inhibit endogenous peroxidase activity. For antigen retrieval, the slides were subjected to microwave-assisted boiling in 1× citrate buffer for 10 min. The slides were incubated with a blocking buffer at room temperature for 1 h to minimize nonspecific antibody binding. The slides were then incubated with an unconjugated anti-human GD3S (1:450) or anti-p53 (1:100) primary antibody for 30 min at room temperature and then incubated with an HRP-conjugated secondary antibody for 30 min at room temperature. A 3,3’-diaminobenzidine substrate solution was then applied to the sections for color development. The slides were counterstained with hematoxylin (Leica Biosystems) for less than 4 min at room temperature and then washed and dehydrated through 4 changes of alcohol (95%, 95%, 100%, 100%) for 5 min each. The slides were then cleared of alcohol in 3 changes of xylene for 5 min each, and coverslips were applied with mounting solution. Images of the slides were acquired using a Vectra Polaris multispectral imager (PerkinElmer). GD3S and p53 expression were quantified with whole-slide digital image analysis using pathology image analysis software from Visiopharm. The expression data were independently validated by a pathologist at MD Anderson.

**Total RNA isolation and gene expression by real-time qPCR**

RNA extraction was performed using a RNeasy Mini Kit (74104; Qiagen) according to the manufacturer’s instructions. For each sample, cDNA was synthesized using 1 μg of RNA and SuperScript IV VILO Master Mix (Invitrogen). Real-time qPCR was performed with the QuantStudio3 system using TaqMan Fast Universal PCR Master Mix (4304437; Applied Biosystems, Thermo Fisher Scientific) as described previously [1]. All samples were run in triplicate. The relative fold changes in specific RNA expression were calculated using the comparative cycle of threshold detection method, and values were normalized to those of glyceraldehyde 3-phosphate dehydrogenase (GAPDH). Fold changes in gene expression were calculated using the 2^–∆∆CT^ method. All primer pairs for human samples (Table S3) were purchased from Thermo Fisher Scientific.

**Chromatin immunoprecipitation (ChIP)-qPCR**

A chromatin extraction kit (ab117152, Abcam) was used to extract chromatin. Cells were crosslinked for 10 min in 1% formaldehyde, quenched in 1.25 M glycine for 5 min at room temperature, washed with ice-cold PBS, and lysed with lysis buffer supplemented with a protease/phosphatase inhibitor cocktail, and the chromatin was pelleted. Chromatin pellets were resuspended in extraction buffer and sheared using a Sonifier SFX150 cell disruptor (Branson Ultrasonics) with a microtip probe (delivering 30-s continuous pulses at 25% power, with 1 min cooling between pulses) for 8 min at 4°C. For immunoprecipitation (IP), a ChIP Kit Magnetic - One Step (ab156907, Abcam) was used. Input lysates were separated from IP lysates, and the IP lysates were incubated for 2 h at room temperature with target and control antibodies loaded on magnetic beads. The magnetic beads were carefully washed, and DNA was eluted from the beads. Input and IP samples were reverse-crosslinked in the DNA release buffer, and chromatin was purified for downstream analysis. The upstream ST8SIA1 promoter region (>2 kb) was fragmented into eleven 240-nucleotide sequences with 60-nucleotide overlaps and designated fragments 1–11 (Fig. 6F). qPCR primer pairs (P1–P11) were designed for each of the 11 nucleotide fragments, and gradient PCR was used to optimize the PCR annealing temperature for each primer pair (Table S4). Real-time qPCR for GD3S was performed using SYBR green (4309155; Applied Biosystems, Thermo Fisher Scientific) in combination with the 11 primer pairs listed in Table S5, and data were presented as the percentage of input DNA. Non-immune IgG was used as the negative control for antibody enrichment and to calculate the percentage of input DNA.

**Luciferase reporter gene assays**

For the assessment of GD3S promoter activity, the ST8SIA1 promoter (human) sequence spanning 2278 bp (2042 bp upstream and 236 bp downstream of the transcription start site) was cloned into the lentivirus promoter reporter plasmid pLL-CMV-Luciferase-T2A-Puro (LL150PA-1, System Biosciences) through the SpeI and NheI restriction enzyme sites and verified with Sanger sequencing. BC cells were plated in a white-walled 96-well plate (Perkin Elmer) at a density of 20,000 cells in 100 μL of growth medium per well. After 24 h, the cells were co-transfected with 100 ng of pLL-ST8SIA1-luciferase-T2A-puro or negative-control Firefly reporter and 50 ng of pLX313-Renilla luciferase (118016; Addgene) constructs using jetPRIME reagent according to the manufacturer’s guidelines. After 24 h of transfection, the cells were treated with dimethyl sulfoxide (DMSO), nutlin-3a (2.5–10 µM), or doxycycline (1 µM) for 24 h, and luciferase activity was detected using the dual-luciferase reporter assay system (E1910; Promega) according to the manufacturer’s instructions. Luminescence was measured using a Spark plate reader (Tecan) at room temperature. To account for variations in transfection efficiency, we normalized Firefly luciferase levels to Renilla luciferase levels to generate a measurement of relative luciferase units that reflects the promoter luciferase activity.

**Tumor spheroid assay**

To assess the impact of doxycycline-induced p53 knockdown on the tumor spheroid–forming ability of different Hs578T and BT549 clones, we seeded SCR_shp53-GD3S_EV, SCR_shp53-GD3S_OE, shp53-GD3S_EV, and shp53-GD3S_OE cells in ultra-low attachment 96-well plates (Corning) at a density of 10 × 10^3^ cells in 100 µL of growth medium. After 3 days, the cells were exposed to either vehicle or 1 µM doxycycline, and spheroid formation and growth were assessed by using the IncuCyte live cell analysis system (Sartorius) to monitor the brightfield area at 6-h intervals for 48–72 h.

**Mammosphere assay**

To investigate the effect of doxycycline-induced p53 knockdown on the mammosphere-forming potential of different Hs578T and BT549 clones, we plated SCR_shp53-GD3S_EV, SCR_shp53-GD3S_OE, shp53-GD3S_EV, and shp53-GD3S_OE cells in ultra-low-attachment 24-well dishes at a density of 5 × 10^3^ cells in 1 mL of mammosphere growth medium (05620; Stemcell Technologies). The cells were treated with vehicle or 1 µM of doxycycline. After 10–12 days of culture, the cells were stained with 1 mg/mL of 3-(4,5-dimethylthiazol-2-yl)-2,5-diphenyltetrazolium bromide (MTT; Alfa Aesar, Thermo Fisher), and mammospheres were counted using the GelCount automated colony counter (Oxford Optronix, Adderbury, United Kingdom) as described previously [1-3].

**Colony formation assay**

The colony formation assay was conducted as described previously [1]. In brief, Hs578T and BT549 cells were seeded in 6-well plates at a density of 1000 cells per well in triplicate. Growth medium (200 µL) containing either vehicle or 1 µM doxycycline was added every 24 h for 2 weeks. The cells were then stained with 1 mg/mL MTT for 2 h at room temperature, and colonies were counted using the GelCount automated colony counter.

**Mitochondrial membrane potential (MMP; ΔΨm) by flow cytometry**

To evaluate mitochondrial membrane polarization, the MitoProbe JC-1 assay kit for flow cytometry (Life Technologies; M34152) was employed. Briefly, a 200 μM JC-1 (5’,6,6’-tetrachloro-1,1’,3,3’-tetraethylbenzimidazolylcarbocyanine iodide, Thermo Fisher Scientific) stock solution was prepared by dissolving the contents of one vial in 230 μL of DMSO. For each sample, 1 × 10^6^ cells were suspended in 1 mL of warm medium or PBS. As a positive control, 1 μL of 50 mM CCCP (carbonyl cyanide 3-chlorophenylhydrazone) was added, and cells were incubated at 37°C for 5 minutes. Subsequently, 10 μL of 200 μM JC-1 (2 μM final concentration) was added, and the cells were incubated at 37°C with 5% CO_2_ for 15 to 30 minutes. The cells were then washed by adding 1 mL of warm PBS and pelleted by centrifugation. Afterward, the cells underwent 1 wash with PBS containing 0.5 μg/mL DAPI and were analyzed on a flow cytometer with 488 nm excitation, using emission filters suitable for Alexa Fluor 488 dye and R-phycoerythrin. Cells were gated to exclude debris and were compensated using the CCCP-treated sample. The data were analyzed by calculating the ratios of red vs. green fluorescence.

**Measurement of oxygen consumption rate in breast cancer cells**

Oxygen consumption was assessed using the Seahorse XF assays with the XFe24 Analyzer from Seahorse Bioscience. The day prior to the assay, the sensor cartridge was immersed in the calibration buffer medium (1 ml) provided by Seahorse Biosciences for overnight hydration. Cells (3.0 × 10^3^) were seeded in V3-PS Seahorse XF96 96-well plates (Agilent) overnight, treated with nutlin-3a (10 µM), doxycycline (1 µM), or vehicle in normal growth medium, and incubated for 6–12 hours. Subsequently, the culture medium was replaced with 500 µl of Seahorse media in each well. After a 1-hour incubation at 37°C in a CO_2_-free atmosphere, basal oxygen consumption rate (OCR) as a respiration indicator and extracellular acidification rate as a glycolysis indicator were measured using the XFe24 analyzer with Seahorse Cell Mito Stress Assay–recommended parameters. The ATP-linked respiration rate was determined by the difference between the initial OCR and the OCR after adding oligomycin (first injection; 1 µM final concentration). Spare respiratory capacity was calculated as the difference between maximum respiration obtained after adding carbonyl cyanide 4-(trifluoromethoxy)phenylhydrazone (FCCP; second injection; 2 µM final concentration) and basal OCR. The mitochondrial respiration rate was determined by the difference between the starting OCR and the OCR after adding an Antimycin A/rotenone mixture (third injection; 1 µM for each drug).

**Immunofluorescence**

The 8-chambered ibidi slides were coated by incubating 300-400 µl of poly-L-lysine (P4832; Millipore Sigma) for 30 minutes at room temperature (RT), followed by a wash in 1× PBS (Corning) and air drying inside a cell-culture incubator. A total of 30-50 × 10^3^ cells were cultured on the 8-chambered slides for 24 hours. MitoTracker Red CMXRos (Thermo Scientific) was prepared by dissolving in 94 µl of sterile DMSO to make a stock concentration of 1 mM. A working concentration of 200 nM was then prepared by dissolving 2 µl from the stock in 10 ml of PBS. Each well received 250 µl of MitoTracker staining buffer, and the slides were incubated for 15–20 minutes in a 37°C CO_2_ incubator. After incubation, the staining buffer was aspirated, and the wells were washed with warm complete medium and then warm PBS. To fix the cells, 250 µl per well of 4% formaldehyde was added for 15 minutes at RT. The cells were then rinsed 3 times with PBS. For permeabilization, 250 µl of 0.25% Triton X-100 was added for 10 minutes or ice-cold acetone for 5 minutes, followed by three washes with PBS. Blocking was performed using 250 µl of 5% FBS in 1× PBS for 30 minutes. For immunostaining, 250 µl of LAMP1 Alexa Fluor 488 (BioLegend), diluted in flow-staining buffer, was added to each well for lysosome staining and incubated at RT for 1 hour. This was followed by 2 washes with PBS. For nuclear staining, 250 µl of 1 µg/ml DAPI was added to each well for 5 minutes, and the wells were washed 2 times with PBS. The chamber bracket was then removed, and a drop of mounting medium was applied to each well, which was then covered using a coverslip (#1.5H; 170 µm ± 5µm). Images were acquired using the Andor revolution spinning disk confocal microscope and analyzed using Fiji software (ImageJ, NIH).

**p53 mutation characterization and expression levels** **in cell lines**

Information pertaining to the types of p53 mutations in each breast cancer cell line was obtained from the TP53 Cell Line Compendium (https://p53.fr/tp53-database/the-tp53-cell-line-compendium) [4]. Cell line p53 mutations were characterized based on type, location, nucleotide, and domain affected (Table S1). Reverse-phase protein array (RPPA)-measured p53 expression levels for each cell line were acquired from the MD Anderson Cell Lines Project (https://tcpaportal.org/mclp/#/) [5, 6].

**RNA sequencing**

**RNA library construction and sequencing:** The extracted RNA was sent to LC Sciences (Houston) for RNA sequencing. Poly(A) RNA sequencing library was prepared following Illumina’s TruSeq-stranded-mRNA sample preparation protocol. RNA integrity was checked with Agilent Technologies 2100 Bioanalyzer. Poly(A) tail-containing mRNAs were purified using oligo-(dT) magnetic beads with two rounds of purification. After purification, poly(A) RNA was fragmented using divalent cation buffer at elevated temperature, followed by DNA library construction. Quality control and quantification of the sequencing library were performed using Agilent Technologies 2100 Bioanalyzer High Sensitivity DNA Chip. Paired-ended sequencing was performed on Illumina’s NovaSeq 6000 sequencing system.

**Pre-processing of RNA-sequencing data:** Cutadapt was used to remove the reads that contained adaptor contamination, low-quality bases, and undetermined bases [7]. After verification of sequencing quality using FastQC (<http://www.bioinformatics.babraham.ac.uk/projects/fastqc/>), HISAT2 was used to map reads to release 107 of the *Homo sapiens* reference genome from Ensemble [8].

The mapped reads of each sample were assembled using StringTie, and all transcriptomes were merged to reconstruct a comprehensive transcriptome using perl scripts and gffcompare. We used StringTie and ballgown (<http://www.bioconductor.org/packages/release/bioc/html/ballgown.html>) to estimate the expression levels of all transcripts [9].

**Differential expression analysis of mRNAs:** Transcripts with average FPKM lower than 1.00 across all 12 samples were removed due to low expression levels, leaving 12,373 unique mRNA transcripts. The 12 samples were equally divided into four groups, each subjected to one condition defined by the combination of genotype (control or overexpression) and compound treatment (DMSO or Nutlin3A). Using the statistic and machine learning toolbox in MATLAB R2021b Update 3, we performed two-way analysis of variance (ANOVA) (balanced design) followed by Tukey’s range tests to identify differentially expressed mRNAs between each pair of conditions. The mRNAs fulfilling all the conditions below were selected for further investigation:

- - p-value≤0.05 for control+DMSO (denoted as CD) vs. control+Nutlin3A (CN) from Tukey’s range tests;
  - p-value≤0.05 for CN vs. overexpression+Nutlin3A (ON) from Tukey’s range tests;
  - Opposite directions of changes for CD-CN and CN-ON, to be consistent with the rescuing effects of the ON condition towards the phenotypes of CN, shown in animal studies;
  - p-value>0.05 for the interaction between genotype and treatment from two-way ANOVA, ensuring the two variables are independent from each other;
  - p-value>0.05 for CD vs. overexpression+DMSO (OD), to be consistent with the phenotypes shown in animal studies.

Ultimately, 74/12,373 (0.595%) mRNAs fulfilled all the conditions above, with 32 genes downregulated by Nutlin3A in control genotypes comparing to DMSO treated baseline (CD>CN), and 42 genes having CD<CN. These transcripts were further filtered by correlation of ST8SIA1 with mitochondrial activity and cell death in TCGA and METABRIC databases, confirming 30/32 genes as a potential panel of pro-survival genes and 22/42 genes as a panel of pro-death genes.

**Pathway analysis for the pro-survival and pro-death group:** Using ConsensusPathDB (Release 35), we carried out over-representation analysis separately on the panel of 32 pro-survival and 22 pro-death genes defined above. Canonical pathways from all 13 resources in that release of ConsensusPathDB were included, namely Biocarta, Ehmn, HumanCyc, Inoh, KEGG, Manual upload, Netpath, Pharmgkb, PID, Reactome, Signalink, SMPDB, and Wikipathways. There were 34 pathways significantly overrepresented (p-value≤0.01) with the 30 pro-survival genes and 19 pathways significantly overrepresented (p-value≤0.01) with the 22 pro-death genes [10, 11].

A 34-by-30 pathway-gene matrix $M={[m_{i,j}]}_{i\in\left[ 1,2\ldots34 \right], j\in\left[ 1,2\ldots30 \right]}$ was generated for the pro-survival panel, where a value 1 for m_(i,j) indicates that the i-th pathway contains the j-th gene as a pathway member. Bi-hierarchical analysis was applied to both rows and columns of matrix M with average linkage and Jaccard index as similarity metric. The clustering and results visualization was done using Morpheus, with number 1’s visualized in green. Similarly, a 22-by-19 matrix was generated and validated for the pro-death panel, with pathway memberships highlighted in red.

Given the comparisons between conditions A and B, each gene’s performances is quantified as performance score ${-log}_{10}\left( p \right)*sign$, with p representing the p-value from Tukey’s range test and sign=1 if condition A is higher than condition B in each of the three comparisons, and sign=-1 otherwise. Three different pre-ranked gene set enrichment analyses were run using the performance scores from three key comparisons (CN-ON, CN-CD, and OD-CD), and a bubble plot was generated using MATLAB R2021b Update 3 summarizing the performance of top 8 pro-survival pathways identified from over-representation analysis.

**Modeling the core protein-protein interaction network responding to the three treatments:** Combining protein products for these 52 genes (30 pro-survival and 22 pro-death) as well as TP53, we obtained 101 protein-protein interactions with combined confidence score≥0.4 based on STRING database v12.0, with 21/52 proteins directly interacting with TP53. Primary localizations for all these proteins were obtained from the Compartments database, showing 12 proteins exclusively localized in the mitochondrion, with 11/12 belonging to the pro-survival panel [12].

The resulting protein-protein interaction networks were visualized in Cytoscape, with each gene’s performances in three different comparisons (CN-ON, CN-CD, and OD-CD) quantified as ${-log}_{10}\left( p \right)*sign$, with p representing the p-value from Tukey’s range test and sign=1 if condition A is higher than condition B in each of the three comparisons (e.g. CN>ON for CN-ON), and sign=−1 otherwise [13]. These three performance scores were visualized as three sectors in one node via the EnhancedGraphics app for Cytoscape [14]. The network is stratified into different sections based on the primary localization information, with the icons for key organelles obtained from Biorender.

**REFERENCES:**

1. Jaggupilli, A., et al., *Metabolic stress induces GD2(+) cancer stem cell-like phenotype in triple-negative breast cancer.* Br J Cancer, 2022. **126**(4): p. 615-627.

2. Ly, S., et al., *Anti-GD2 antibody dinutuximab inhibits triple-negative breast tumor growth by targeting GD2(+) breast cancer stem-like cells.* J Immunother Cancer, 2021. **9**(3).

3. Nguyen, K., et al., *ST8SIA1 Regulates Tumor Growth and Metastasis in TNBC by Activating the FAK-AKT-mTOR Signaling Pathway.* Mol Cancer Ther, 2018. **17**(12): p. 2689-2701.

4. Berglind, H., et al., *Analysis of p53 mutation status in human cancer cell lines: a paradigm for cell line cross-contamination.* Cancer Biol Ther, 2008. **7**(5): p. 699-708.

5. El-Dana, F., et al., *Abstract PS16-19: Hotspot p53 mutations correlate with increased expression of stem cell markers in triple-negative breast cancer.* Cancer Research, 2021. **81**(4_Supplement): p. PS16-19-PS16-19.

6. Li, J., et al., *Characterization of Human Cancer Cell Lines by Reverse-phase Protein Arrays.* Cancer Cell, 2017. **31**(2): p. 225-239.

7. Martin, M., *Cutadapt removes adapter sequences from high-throughput sequencing reads.* 2011, 2011. **17**(1): p. 3.

8. Kim, D., B. Langmead, and S.L. Salzberg, *HISAT: a fast spliced aligner with low memory requirements.* Nat Methods, 2015. **12**(4): p. 357-60.

9. Pertea, M., et al., *StringTie enables improved reconstruction of a transcriptome from RNA-seq reads.* Nat Biotechnol, 2015. **33**(3): p. 290-5.

10. Curtis, C., et al., *The genomic and transcriptomic architecture of 2,000 breast tumours reveals novel subgroups.* Nature, 2012. **486**(7403): p. 346-52.

11. Herwig, R., et al., *Analyzing and interpreting genome data at the network level with ConsensusPathDB.* Nat Protoc, 2016. **11**(10): p. 1889-907.

12. Binder, J.X., et al., *COMPARTMENTS: unification and visualization of protein subcellular localization evidence.* Database (Oxford), 2014. **2014**: p. bau012.

13. Shannon, P., et al., *Cytoscape: a software environment for integrated models of biomolecular interaction networks.* Genome Res, 2003. **13**(11): p. 2498-504.

14. Morris, J.H., et al., *enhancedGraphics: a Cytoscape app for enhanced node graphics.* F1000Res, 2014. **3**: p. 147.
